# Supplementary material for: Docking for EP4R antagonists active against inflammatory pain
Source: Nat Commun. 2023 Dec 6;14:8067. doi: 10.1038/s41467-023-43506-6 (PMC10700596; doi:10.1038/s41467-023-43506-6)
Supplement: Supplementary file 1 — Supplementary Information [file 41467_2023_43506_MOESM1_ESM.pdf]

Supplementary Information

Docking for Structure-based Design of Anti-inflammatory EP4R Antagonists

Stefan Gahbauer[1,†], Chelsea DeLeon[2,†], Joao Braz[3,†], Veronica Craik[3], Hye Jin Kang[2,§], Xiaobo Wan[1], Xi-Ping Huang[2], Christian B Billesbølle[1], Yongfeng Liu[2], Tao Che[2,#], Ishan Deshpande[1], Madison Jewell[3], Elissa A Fink[1], Ivan S Kondratov[4,5], Yurii S Moroz[6,7], John J Irwin[1], Allan I Basbaum[3,*], Bryan L Roth[2,8,9,*] & Brian K Shoichet[1,*]

[1]Department of Pharmaceutical Chemistry, University of California San Francisco, San Francisco, CA 94158, USA

[2]Department of Pharmacology, University of North Carolina at Chapel Hill School of Medicine, Chapel Hill, NC 27514, USA

[3]Department of Anatomy, University of California, San Francisco, San Francisco, CA 94158, USA

[4]Enamine Ltd. Kyiv, Ukraine

[5]V.P. Kukhar Institute of Bioorganic Chemistry and Petrochemistry, National Academy of Sciences of Ukraine, Kyiv, Ukraine

[6]Chemspace LLC, Kyiv, Ukraine

[7]National Taras Shevchenko University of Kyiv, Kyiv, Ukraine

[8]National Institute of Mental Health Psychoactive Drug Screening Program, University of North Carolina at Chapel Hill School of Medicine, Chapel Hill, NC 27514, USA

[9]Division of Chemical Biology and Medicinal Chemistry, University of North Carolina at Chapel Hill Eshelman School of Pharmacy, Chapel Hill, NC 27514, USA

[§]Present address: Department of Biotechnology, College of Life Science and Biotechnology, Yonsei University, Seoul, South Korea

[#]Present address: Center of Clinical Pharmacology, Department of Anesthesiology, Washington University School of Medicine, St. Louis, MO 63110, USA

[†] These authors contributed equally.

* Corresponding authors. Email: bryan_roth@med.unc.edu (B.L.R.), allan.basbaum@ucsf.edu (A.I.B), bshoichet@gmail.com (B.K.S.)

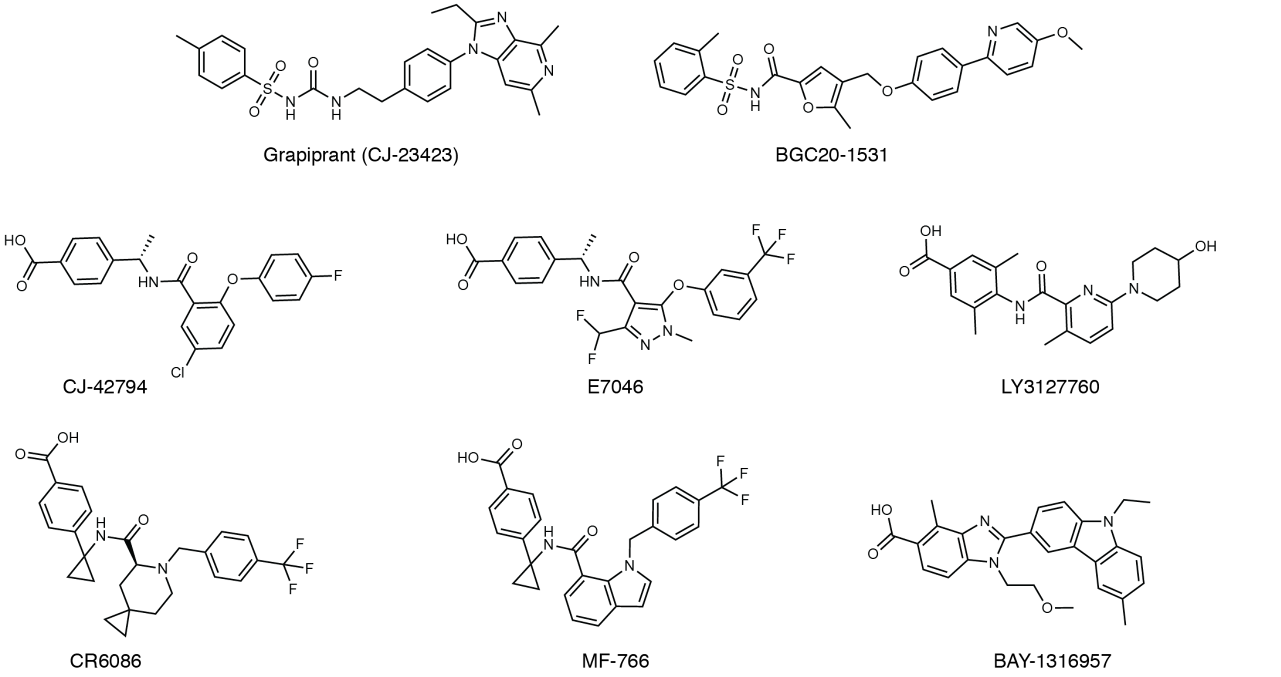

Supplementary Figure 1: Representative previously reported EP4R antagonists.

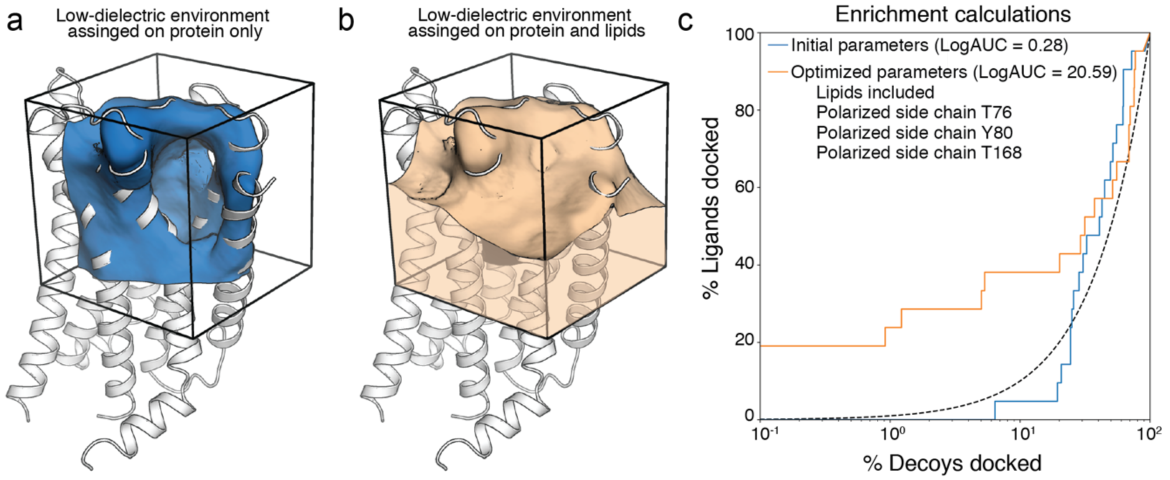

Supplementary Figure 2: Optimization of DOCK3.7 scoring functions. **a)** The low-dielectric environment (relative dielectric constant $\varepsilon_r = 2$), shown as a blue surface, e.g. for the ligand desolvation scoring function, is by default generated on protein atoms only as the protein is assumed to be solvated by water. **b)** The low-dielectric environment, shown as an orange surface, was generated on protein and lipid atoms (not shown for clarity) derived from molecular dynamics simulations and expands beyond the boundaries of the protein within the scoring box. **c)** Enrichment of 21 previously reported EP4 receptor antagonists against a background of 658 decoys was assessed by computing the area under the semilogarithmic receiver-operator characteristic curve (LogAUC). The optimized scoring functions (orange), reflecting the protein-lipid system and polarized side chain hydroxyl groups of Thr76, Tyr80 and Thr168, achieved a meaningfully higher LogAUC than the default scoring terms (blue).

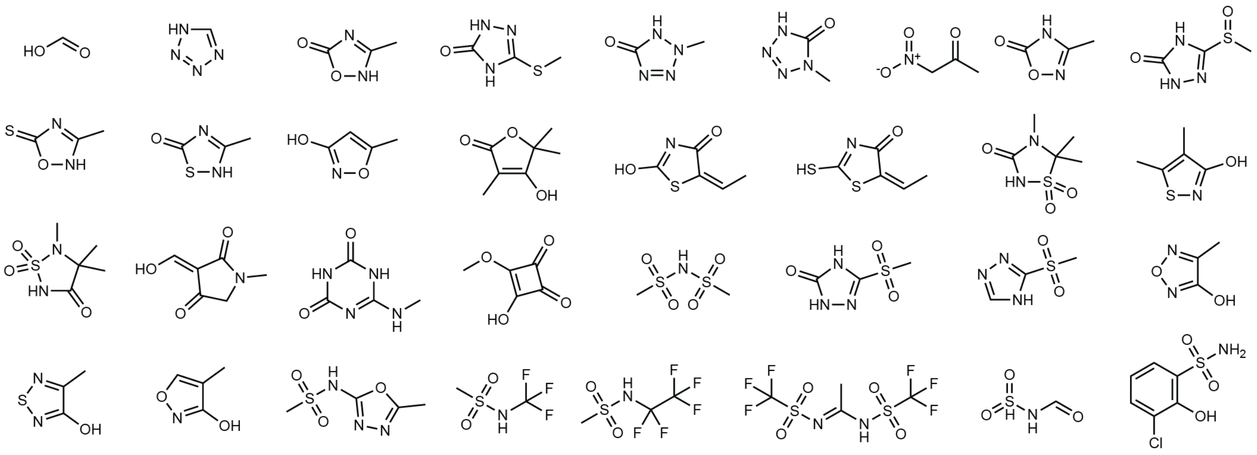

Supplementary Figure 3: Functional groups used for searching anionic compounds in the Enamine REAL space.

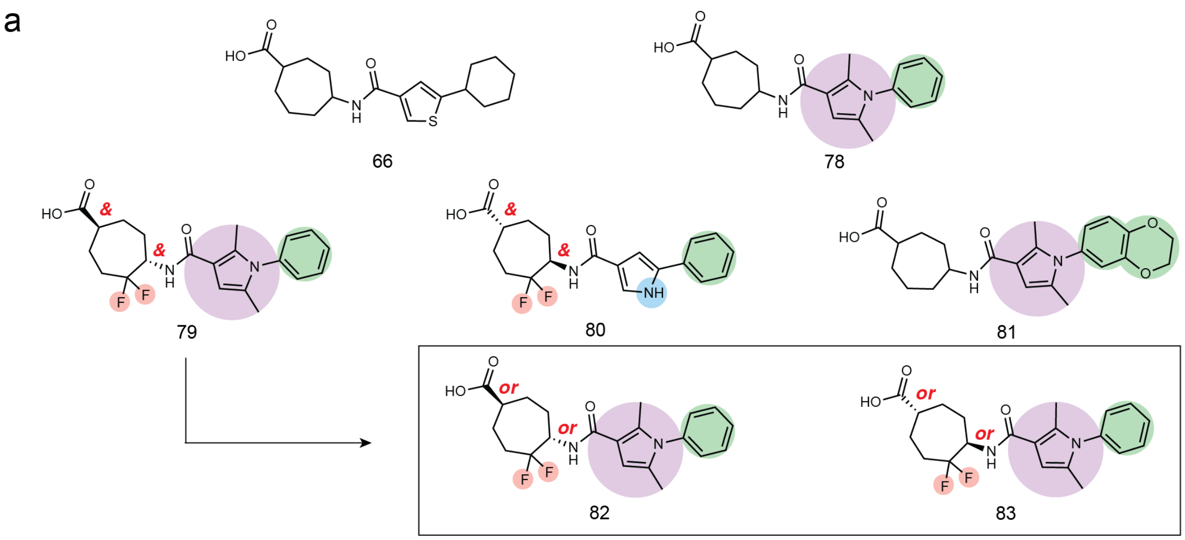

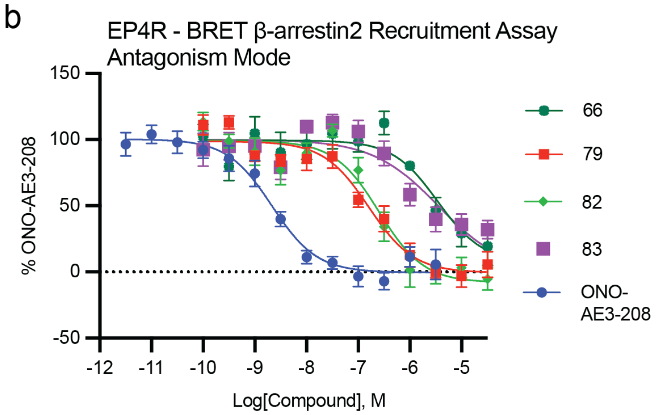

c

Compound	K_i (nM)
66	810
79	32
82	44
83	>10,000

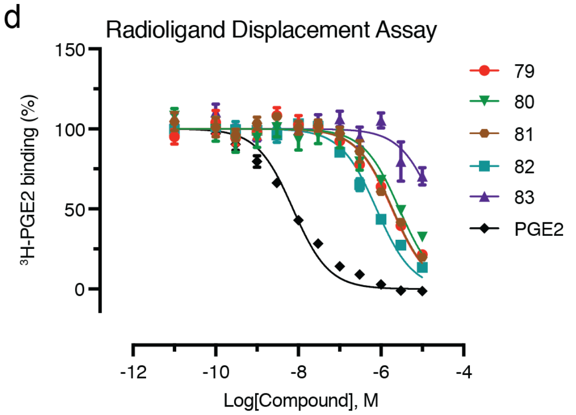

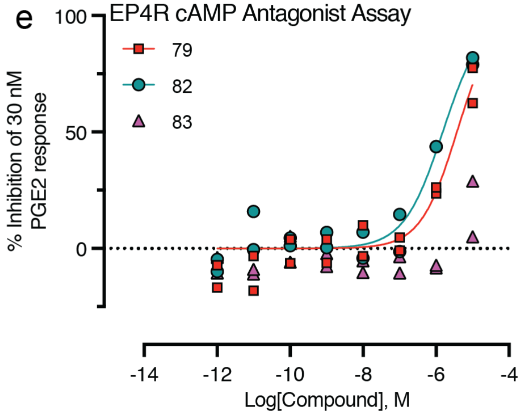

Supplementary Figure 4: Optimization of the docking hit 66. **a)** Chemical structures of 66 and analogs with improved potencies. "*&*" denotes stereomeric mixtures, i.e. compounds were tested as mixtures of two enantiomers. "*or*" denotes pure enantiomers, however, the absolute stereochemistry was not determined. **b)** Dose response curves for compounds shown in a) obtained from BRET-based EP4R-arrestin interaction reporter assays. **c)** Calculated Ki values using the Cheng-Prusoff equation. **d)** Radioligand (^{3}H-PGE2) displacement assays at EP4R. **e)** EP4 functional cyclic AMP production assay (Eurofins item 1872). Data in b) and d) represents mean ± SEM of at three independent experiments. Data in e) represents two technical repetitions. Source data are provided as a Source Data file.

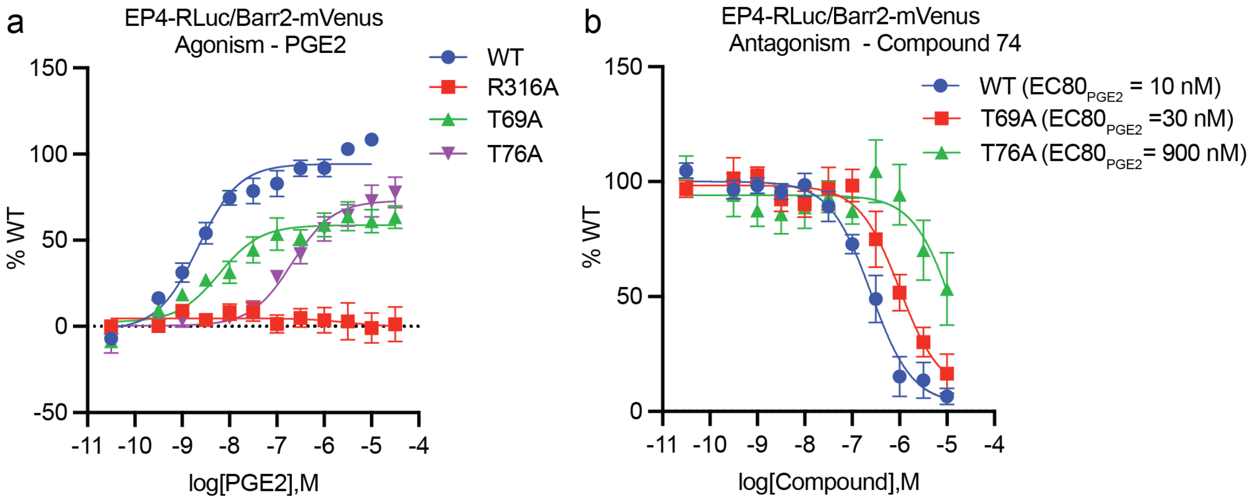

c

	EC50 [nM] (PGE2)	EC80 [nM] (PGE2)
WT	2.2	10
T69A	6.1	30
T76A	210	900
R316A	-	-

d

	IC50 [µM] (74)
WT	0.2
T69A	1.0
T76A	14.7
R316A	-

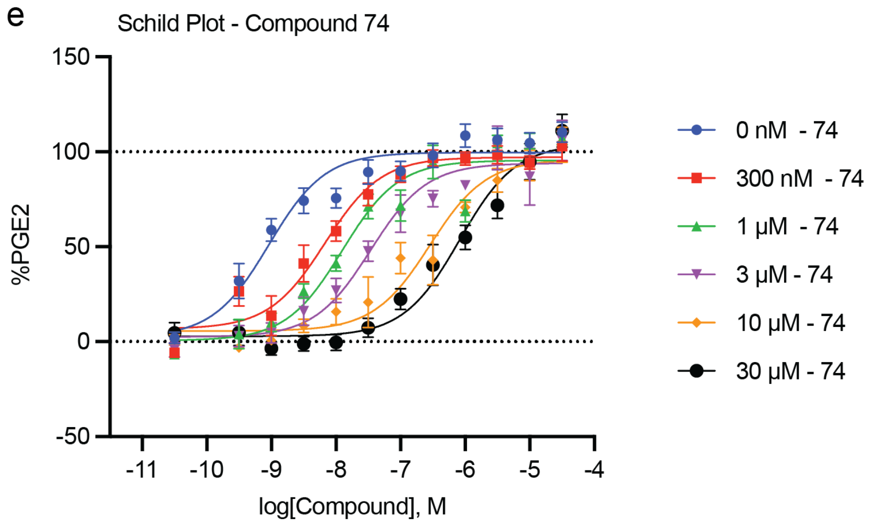

Supplementary Figure 5: Mutagenesis supports predicted binding pose of 74. **a)** BRET-based EP4R-arrestin interaction reporter assay for WT EP4R as well as R316A, T69A and T76A mutations for EC50/80 determination of PGE2 and **b)** for measuring IC50 of 74. For running in antagonist mode, PGE2 was added according to the mutant-specific EC80 concentration. **c)** EC50 and EC80 of PGE2 at WT and mutant EP4R. **d)** IC50 of 74 at WT and mutant EP4R. **e)** Schild Plot of 74. Data in a), b), e) represent mean ± SEM of three independent experiments. Source data are provided as a Source Data file.

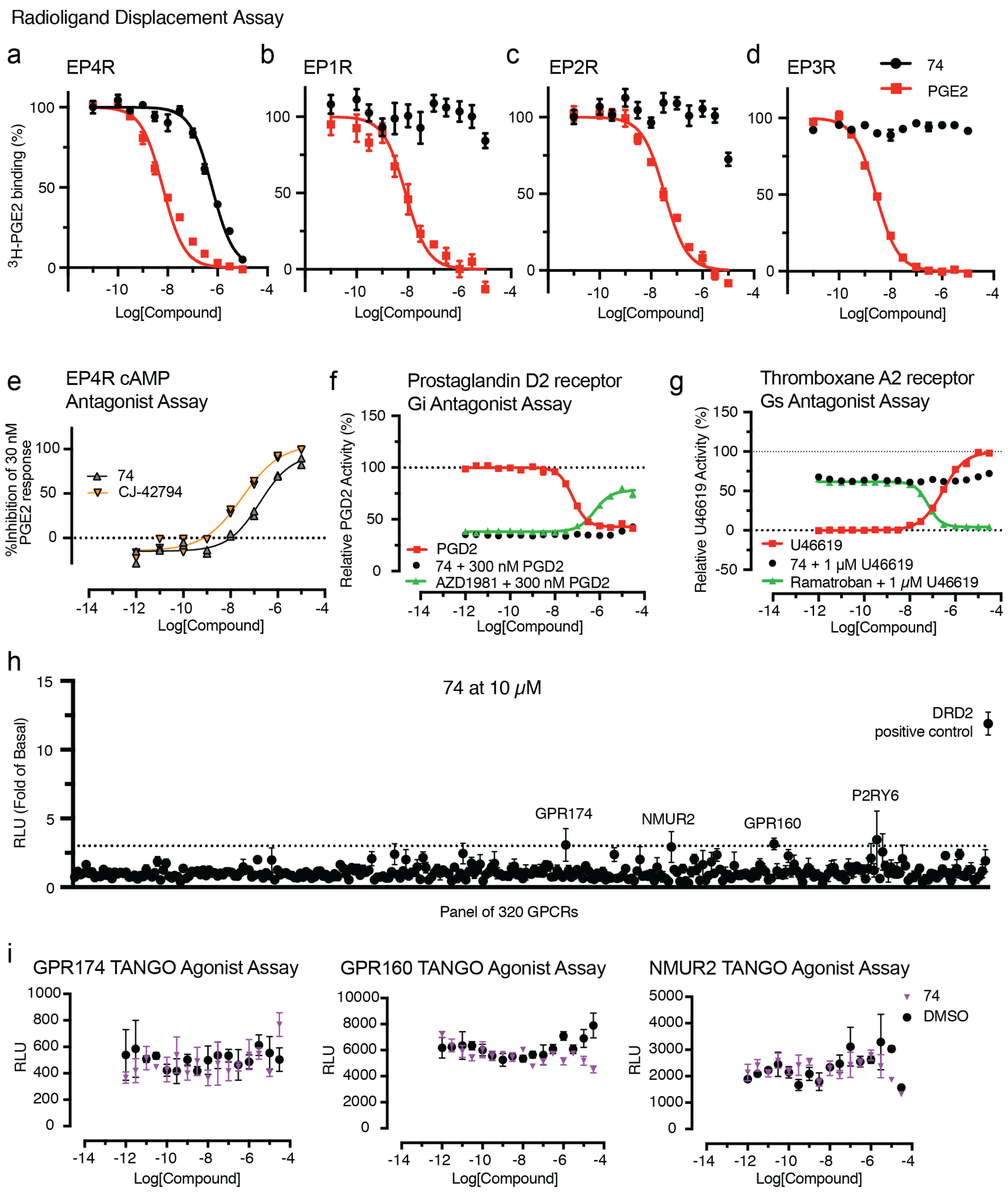

Supplementary Figure 6: GPCR pharmacology of 74. **a)-d)** Radioligand (^{3}H-PGE2) displacement assays at EP4R, EP1R, EP2R and EP3R respectively. 74 obtained a K_i of 366 nM, PGE2 of 3.5 nM at EP4R. **e)** EP4R functional cyclic AMP production assay. Dose-response curves were obtained for 74 (K_B = 16 nM) and CJ-42794 (K_B = 2.9 nM) against 30 nM PGE2 (Eurofins item 1872). **f), g)** Functional cAMP production assays for the prostaglandin D2 (PGD2) receptor or the Thromboxane A2 (TP) receptor, respectively. AZD1981 and Ramatroban were used as control PGD2 and TP receptor antagonists, respectively. U46619 served as a TP receptor agonist. **H)** GPCRome off-target screening of 74 at 10 μM. **i)** Potential off-targets that met the activity threshold (3-fold of increase of basal) in the GPCRome screen (GPR174, GPR160, or NMUR2) were investigated with full dose-response curves in PRESTO-Tango assays and showed no activity. Data in a)-d), f)-g), i) represents mean ± SEM of three independent experiments. Data in e) represents two technical replicates. Data in h) represents mean ± SEM of four technical replicates. Source data are provided as a Source Data file.

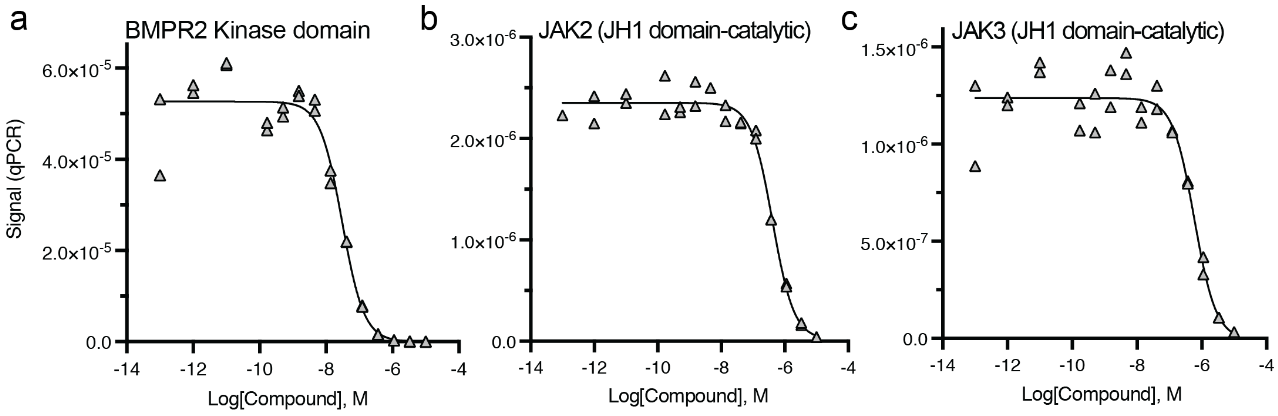

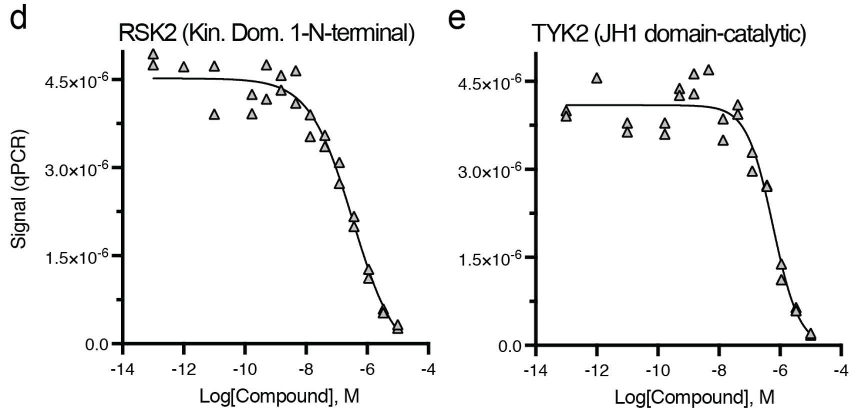

f

Target	74 K_d (nM)
BMPR2	30
JAK2	410
JAK3	560
RSK2	310
TYK2	580

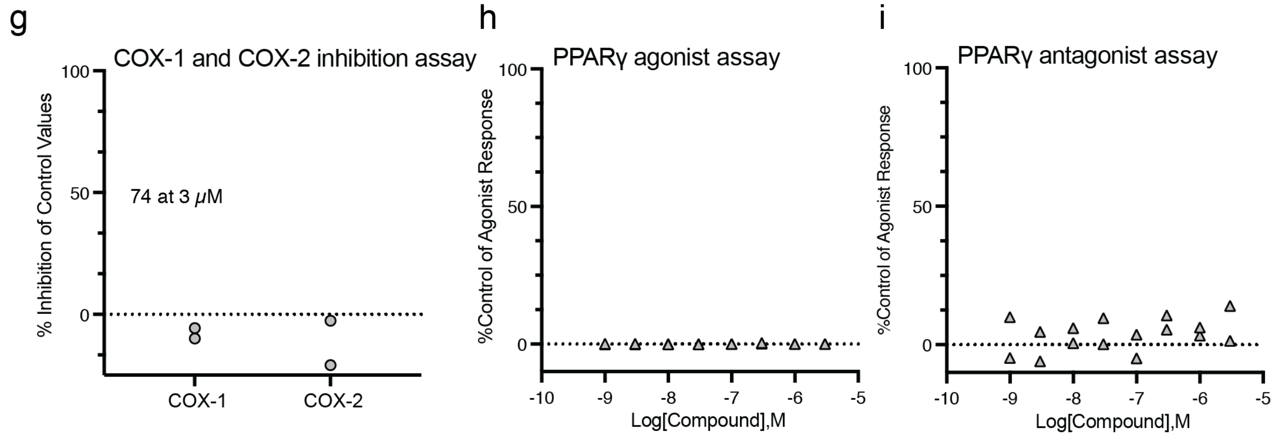

Supplementary Figure 7: Non-GPCR off-target screening for 74. **a)-e)** Dose-response curves of 74 at kinase hits from the single-concentration *scanEDGE* Kinase Panel of 97 kinases using DiscoverX *KINOMEscan* (Eurofins item 87-0002-1000, see Supplementary Data set 2). **f)** Calculated K_d values of 74 at five kinases. **g)** 74 showed no off-target activity COX-1 and COX-2 at 3 μM (Eurofins items 4173 and 4186, respectively). **h)-i)** 74 showed no agonist or antagonist activity at the nuclear hormone peroxisome proliferator-activated receptor gamma (PPARγ, Eurofins item G124). Data in a)-e) and g)-i) represent two technical replicates. Source data are provided as a Source Data file.

	Grapiprant				CJ-42794				74				77				82			
	2 mg/ml		5 mg/ml		2 mg/ml		5 mg/ml		2 mg/ml		5 mg/ml		2 mg/ml		5 mg/ml		2 mg/ml		5 mg/ml	
Vehicle	3 h	24 h	3 h	24 h	3 h	24 h	3 h	24 h	3 h	24 h	3 h	24 h	3 h	24 h	3 h	24 h	3 h	24 h	3 h	24 h
2HPβ-cyclodextrin - Saline (20%:80%)	-	-	D	D	ND	ND	ND	ND	-	-	D	D	-	-	D	D	-	-	D	D
Captisol - Water (40%:60%)	-	-	-	-	ND	ND	ND	ND	-	-	-	-	-	-	-	-	-	-	-	-
Captisol - Saline - Water (20%:40%:40%)	-	-	D	D	ND	ND	ND	ND	-	-	D	D	-	-	D	D	-	-	D	D
Kolliphor EL - Water (20%:80%)	-	-	D	D	ND	ND	ND	ND	ND	ND	ND	ND	-	-	D	D	-	-	D	D
Kolliphor HS15 - Saline - Water (20%:40%:40%)	-	-	D	D	D	D	ND	ND	ND	ND	ND	ND	-	-	D	D	-	-	D	D
DMSO - PEG400 - Water (20%:40%:40%)	-	-	D	D	ND	ND	ND	ND	-	-	D	ND	ND	ND	ND	ND	-	-	D	D
DMSO - Kolliphor EL: 5% Mannitol in Water 1:8 (10%:90%)	-	-	-	-	D	ND	D	ND	-	-	D	ND	-	-	D	D	-	-	D	D
Ethanol - Glycerol - Water (10%:20%:70%)	-	-	-	-	ND	ND	ND	ND	-	-	D	ND	ND	ND	ND	ND	-	-	D	D
DMA - PEG400 - Water (20%:20%:60%)	-	-	-	-	ND	ND	ND	ND	-	-	D	ND	ND	ND	ND	ND	-	-	ND	ND
Dextran 40 - Saline (10%:90%)	-	-	-	-	-	-	-	-	-	-	D	ND	ND	ND	ND	ND	-	-	-	-

Supplementary Figure 8: Formulation screen for *in vivo* experiments. “D” indicates the compound dissolved, while “ND” denotes the compound did not remain in solution.

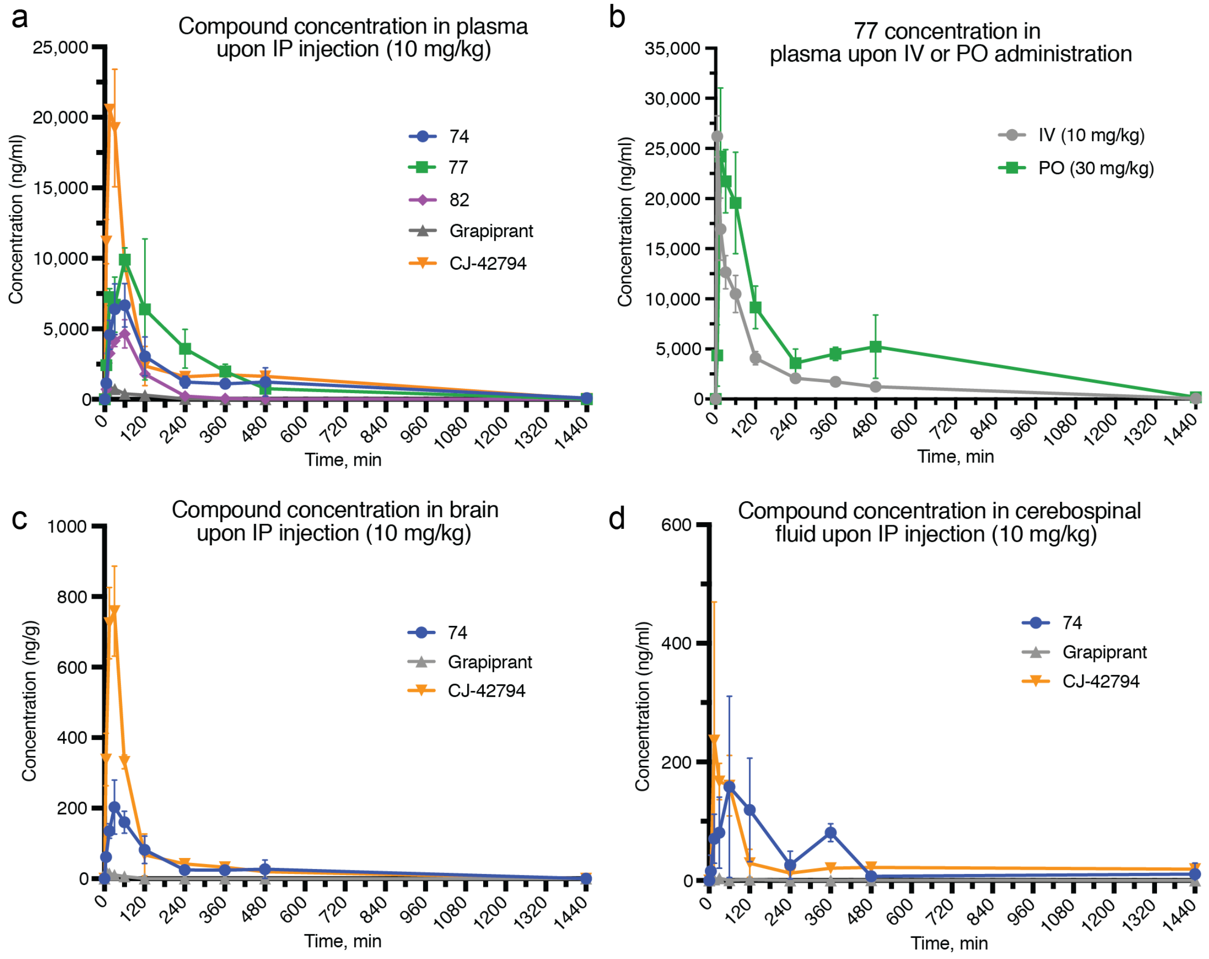

e

	Plasma						Brain					Cerebospinal fluid				
Compound	T_{max} [min]	C_{max} [ng/ml]	C_{max}* [μM]	$AUC_{0-\infty}$ [ng min/ml]	$T_{1/2}$ [min]	F [%]	T_{max} [min]	C_{max} [ng/g]	C_{max}* [μM]	$AUC_{0-\infty}$ [ng min/g]	$T_{1/2}$ [min]	T_{max} [min]	C_{max} [ng/ml]	C_{max}* [μM]	$AUC_{0-\infty}$ [ng min/ml]	$T_{1/2}$ [min]
74	60	6,670	18.12	1,450,000	179	-	30	203	0.55	34,300	142	30	158	0.42	35,000	115
77	60	9,910	26.90	2,370,000	156	78	-	-	-	-	-	-	-	-	-	-
82	60	4,640	11.89	551,000	59.3	-	-	-	-	-	-	-	-	-	-	-
Grapiprant	15	834	1.79	80,600	48.6	5†	5	21.9	0.04	1,080	39.2	5	9,31	0.02	268	26.2
CJ-42794	15	20,600	49.78	2,720,000	176	73‡	30	760	1.84	65,200	210	15	236	0.57	212,000	6,230

Supplementary Figure 9: Pharmacokinetic profiles of EP4R antagonists. **a)** Compound concentrations in plasma upon 10 mg/kg intraperitoneal (i.p.) injections in mice. **b)** Concentrations of 77 in plasma upon 10 mg/kg intravenous (i.v.) injections or 30 mg/kg oral administration in mice. **c)** Compound concentrations in brain upon 10 mg/kg i.p. injections in mice. 77 and 82 were excluded from this experiment. **d)** Compound concentrations in cerebrospinal fluid (CSF) upon 10 mg/kg i.p. injections in mice. 77 and 82 were excluded from this experiment. **e)** Pharmacokinetic parameters. C_{max}* was calculated by dividing the measured C_{max} by the compounds' molecular weights. We note that the accuracy of the calculated parameters in the CSF is limited due to the low exposure of the compounds in the brain and CSF. †, ‡ The oral bioavailability values of Grapiprant and CJ-42794 were previously described.[1,2] Data in a)-d) represents mean ± SEM of three animals per time point. Source data are provided as a Source Data file.

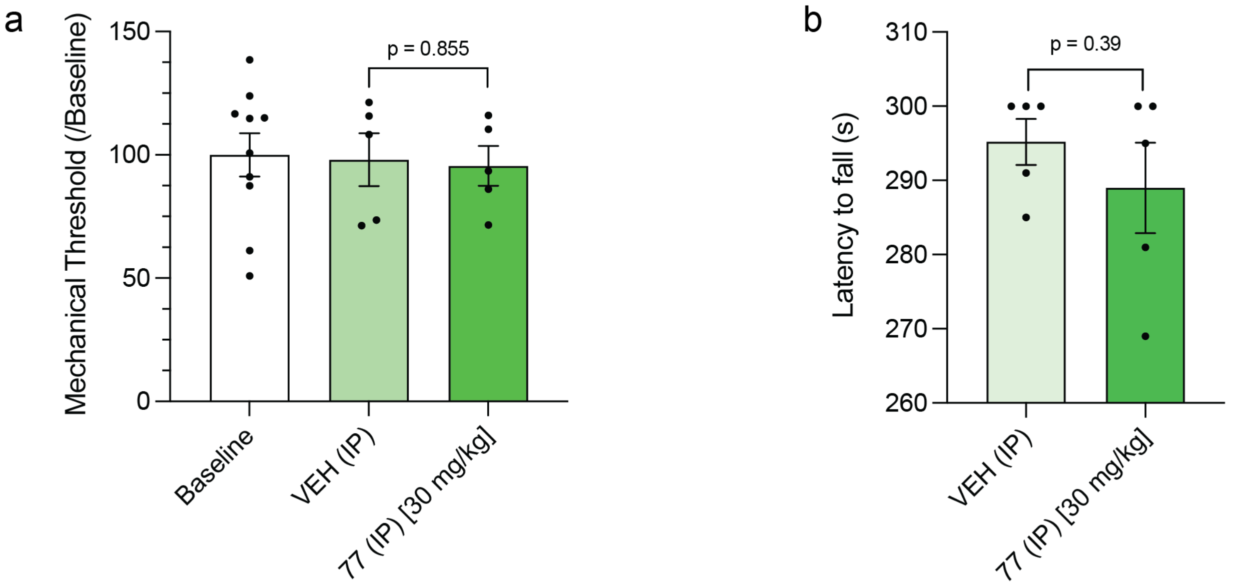

Supplementary Figure 10: In vivo characterization of 77 in uninjured mice. "n" denotes number of independent animals. **a)** 77 did not modulate mechanical thresholds in uninjured mice (n = 5 in vehicle and treated group). **b)** 77 did not show significant motor-impairment upon intraperitoneal administration in the rotarod test (n = 5). *p*-values between indicated groups were calculated with two-tailed unpaired *t*-tests. In a), b) data is shown as mean ± SEM. Source data are provided as a Source Data file.

Supplementary Methods

General synthetic procedures and chemical identities.

Method 1. An amine (100 mg), an acid (1.1 mol. eq. to the amine), and 0.5 mL of DMSO were placed into a 4 mL capped glass vial and the mixture was stirred for 30 min. Then **1**-Ethyl-3-(3-dimethylaminopropyl)carbodiimide (EDC, 1.2 mol. eq. to the amine) was added and the mixture was stirred for 1 hour. If the solution was transparent, the mixture was left overnight at room temperature as is. Otherwise, the vial was placed in the ultrasonic bath and left overnight. The solution was filtered, and the solvent and volatile components were evaporated under reduced pressure to give the crude product. The product was further purified by HPLC.

Method 2. A monoester of dicarboxylic acid (100 mg), 1% hydroxybenzotriazole (HOBt) in DMF, an amine (1 mol. eq. to the monoester of dicarboxylic acid), and triethanolamine (TEA, 1 mol. eq. to the amine in case the latter is used as a salt) were place in a vial with a screw cap. The vial was left shaking for 24 hours at room temperature and chloroform (3 mL) and water (1 mL) were added. After 15 min, the organic phase was separated and dried over Na_2SO_4. Then, DMSO (0.5 mL) was added, and the solution was left shaking overnight at room temperature followed by the addition of 4M KOH (2 mol. eq. to the monoester of dicarboxylic acid). The solution was shaken for 12 hours at room temperature and further neutralized with formic acid. The solvent and volatile components were evaporated under reduced pressure to give the crude product. The product was further purified by HPLC.

Method 3. An amine (100 mg), DIPEA (1.2 mol equivalent to the amine), and DMSO (0.5 mL) were placed into a 4 mL capped glass vial and stirred for 30 min. After the addition of an aryl halide (1.2 mol eq. to the amine), the mixture was stirred for one hour at room temperature. Then the vial was placed in an oven (set

to 100°C) for 9 hours. After cooling down the mixture was filtered; the solvent and volatile components were evaporated under reduced pressure to give the crude product. The product was further purified by HPLC.

Method 4. Methylene-active compound (100mg), aldehyde (1 mol eq. to the methylene-active compound), DMF (0.5 mL), and trimethylchlorosilane (2.1 mol eq. to the methylene-active compound) were placed in the vial. The vial was then kept in an oven (set to 100°C) for 48 hours. After cooling the reaction mixture down to room temperature, DIPEA (0.2 mL) was added to it, and the solution was stirred for 30 min. The solvents were evaporated under reduced pressure to give a crude product. The product was further purified by HPLC.

Method 5. An amino acid ester (100 mg), an aryl halide (1 mol eq. to the ester), N,N-diisopropylethylamine (DIPEA, 1 mol. eq. to the amine or 2 mol eq., in case the latter is used as salt) are dissolved in 0.5 mL of DMSO in a vial with a screw cap. The vial was shaken at room temperature for 1 hour followed by keeping it in an oven overnight at 135°C. After cooling down to room temperature, 2 mL of methanol was added and the solution was shaken for 1 hour. The solvent was evaporated under reduced pressure. 4M KOH (0.4 mL of aqueous solution) was added to the reaction mixture. The vial was shaken for 12 hours and the solution was neutralized with formic acid (0.1 mL). The solvent was further evaporated and the crude sample was purified by HPLC.

Spectral Description.

4-(2-(tert-butyl)thiazole-4-carboxamido)cycloheptane-1-carboxylic acid – 103, 4899_664, Z3509940713 **(Method 2).**
Yield: 54%; purity, >95% (assessed by LC/MS).
^{1}H NMR (600 MHz, DMSO-d_6) δ 12.01 (s, 1H), 8.06 (s, 1H), 7.82 (d, *J* = 8.6 Hz, 1H), 3.91 – 3.84 (m, 1H), 2.46 – 2.40 (m, 1H), 1.93 – 1.84 (m, 2H), 1.81 (s, 1H), 1.86 – 1.76 (m, 1H), 1.68 (dddd, *J* = 17.2, 13.8, 8.3, 4.7 Hz, 1H), 1.65 – 1.47 (m, 5H), 1.39 (s, 9H).
LC/MS (APSI) m/z [M+H] calculated for C16H25N2O3S: 325.2; found: 325.2.

2-(4-(1-(p-tolyl)-1,4,5,6-tetrahydrocyclopenta[c]pyrazole-3-carboxamido)phenyl)acetic acid – 64, Z1664425040, Z1664425040 **(Method 2).**
Yield: 48%; purity, >95% (assessed by LC/MS).
^{1}H NMR (500 MHz, DMSO-d_6) δ 12.26 (s, 1H), 9.89 (s, 1H), 7.72 (dd, *J* = 16.1, 8.0 Hz, 4H), 7.32 (d, *J* = 8.0 Hz, 2H), 7.20 (d, *J* = 8.1 Hz, 2H), 3.51 (s, 2H), 3.02 (t, *J* = 7.3 Hz, 2H), 2.76 (t, *J* = 7.2 Hz, 2H), 2.57 (p, *J* = 7.3 Hz, 2H), 2.34 (s, 3H).
LC/MS (APSI) m/z [M+H] calculated for C22H22N3O3: 376.2; found: 376.2.

4-(4-(4-fluorophenyl)-5-methylthiophene-2-carboxamido)cycloheptane-1-carboxylic acid – 108, 4899_735, Z4658412026 **(Method 2).**
Yield: 42%; purity, >95% (assessed by LC/MS).
^{1}H NMR (600 MHz, DMSO-d_6) δ 12.01 (s, 1H), 8.15 (d, *J* = 7.9 Hz, 1H), 7.80 (s, 1H), 7.50 – 7.43 (m, 2H), 7.28 (t, *J* = 8.8 Hz, 2H), 3.85 (s, 1H), 2.44 (s, 3H), 2.41 (s, 1H), 1.91 – 1.86 (m, 2H), 1.86 – 1.77 (m, 1H), 1.71 – 1.58 (m, 2H), 1.50 (ddt, *J* = 18.1, 15.2, 7.2 Hz, 4H).
LC/MS (APSI) m/z [M+H] calculated for C20H23FNO3S: 376.2; found: 376.2.

3-(5-cyclohexylthiophene-3-carboxamido)-1-methylcyclobutane-1-carboxylic acid – 111, 4899_1086, Z4658411999 **(Method 2).**
Yield: 23%; purity, >95% (assessed by LC/MS).
^{1}H NMR (400 MHz, DMSO-d_6) δ 12.31, 8.37, 8.34, 8.32, 7.88, 7.86, 7.25, 7.22, 4.45, 4.42, 4.40, 4.38, 4.36, 3.33, 2.77, 2.66, 2.64, 2.63, 2.61, 2.50, 2.41, 2.13, 2.11, 2.09, 2.02, 2.00, 1.99, 1.97, 1.77, 1.75, 1.69, 1.65, 1.42, 1.39, 1.36, 1.34, 1.32, 1.21, 1.18.
LC/MS (APSI) m/z [M+H] calculated for C17H24NO3S: 322.2; found: 322.2.

rel-(1R,5R)-4,4-difluoro-5-(1-(4-fluorophenyl)-2,5-dimethyl-1H-pyrrole-3-carboxamido)cycloheptane-1-carboxylic acid – 130, 1757_0010, Z4906033314 **(Method 2).**
Yield: 76%; purity, >95% (assessed by LC/MS).
^{1}H NMR (500 MHz, DMSO-d_6) δ 12.27 (s, 1H), 7.50 (d, *J* = 9.3 Hz, 1H), 7.41 – 7.28 (m, 4H), 6.45 (s, 1H), 4.40 (ddd, *J* = 19.6, 10.3, 3.6 Hz, 1H), 2.17 (s, 3H), 2.14 (s, 1H), 2.14 – 1.97 (m, 2H), 1.91 (s, 4H), 1.80 – 1.54 (m, 4H).
LC/MS (APSI) m/z [M+H] calculated for C21H24F3N2O3: 409.2; found: 409.2.

rel-(1R,5R)-4,4-difluoro-5-(1-(3-fluorophenyl)-2,5-dimethyl-1H-pyrrole-3-carboxamido)cycloheptane-1-carboxylic acid – 128, 1757_0011, Z4906033318 **(Method 2).**
Yield: 19%; purity, >95% (assessed by LC/MS).
^{1}H NMR (600 MHz, DMSO-d_6) δ 12.21 (s, 1H), 7.57 (q, *J* = 7.9 Hz, 1H), 7.51 (d, *J* = 9.3 Hz, 1H), 7.34 (td, *J* = 8.6, 2.6 Hz, 1H), 7.26 (dt, *J* = 9.6, 2.3 Hz, 1H), 7.13 (dd, *J* = 7.9, 1.9 Hz, 1H), 6.46 (s, 1H), 4.41 (ddd, *J* = 19.4, 10.1, 3.6 Hz, 1H), 2.20 (s, 3H), 2.15 (s, 2H), 2.13 – 1.97 (m, 2H), 1.94 (s, 3H), 1.92 – 1.82 (m, 1H), 1.68 (dddd, *J* = 51.1, 25.3, 11.8, 8.6 Hz, 3H), 1.67 (s, 2H).
LC/MS (APSI) m/z [M+H] calculated for C21H24F3N2O3: 409.2; found: 409.4.

rel-(1R,5R)-5-(2,5-dimethyl-1-phenyl-1H-pyrrole-3-carboxamido)-4,4-difluorocycloheptane-1-carboxylic acid – 79, 1757_0003, Z4906033303 **(Method 2).**
Yield: 25%; purity, >95% (assessed by LC/MS).
^{1}H NMR (400 MHz, DMSO-d_6) δ 12.23 (s, 1H), 7.53 (dt, *J* = 16.5, 6.7 Hz, 5H), 7.33 – 7.22 (m, 3H), 6.47 (s, 1H), 4.44 (s, 1H), 2.54 (s, 1H), 2.19 (s, 4H), 2.17 – 1.98 (m, 1H), 1.93 (s, 4H), 1.87 (s, 1H), 1.82 – 1.57 (m, 3H).
LC/MS (APSI) m/z [M+H] calculated for C21H25F2N2O3: 391.2; found: 391.2.

rel-(1R,5R)-5-(2,5-dimethyl-1-phenyl-1H-pyrrole-3-carboxamido)-4,4-difluorocycloheptane-1-carboxylic acid – 82, Z4989072931, Z4989072931 **(Method 2).**
Yield: 82%; purity, >95% (assessed by LC/MS).
^{1}H NMR (600 MHz, DMSO-d_6) δ 12.20 (s, 1H), 7.53 (t, *J* = 7.6 Hz, 2H), 7.48 (t, *J* = 7.7 Hz, 2H), 7.26 (dd, *J* = 7.5, 1.6 Hz, 2H), 6.45 (s, 1H), 4.41 (dd, *J* = 19.5, 9.7 Hz, 1H), 2.17 (s, 3H), 2.13 (s, 2H), 2.22 -1.99 (m, 2H), 1.91 (s, 3H), 1.87 (s, 1H), 1.92 – 1.81 (m, 0H), 1.80 – 1.63 (m, 2H), 1.67 (s, 2H), 1.68 – 1.56 (m, 1H).
LC/MS (APSI) m/z [M+H] calculated for C21H25F2N2O3: 391.2; found: 391.2.

rel-(1R,5R)-4,4-difluoro-5-(1-(2-fluorophenyl)-2,5-dimethyl-1H-pyrrole-3-carboxamido)cycloheptane-1-carboxylic acid – 113, 1757_0012, Z4906033323 **(Method 2).**
Yield: 67%; purity, >95% (assessed by LC/MS).
^{1}H NMR (600 MHz, DMSO-d_6) δ 8.44 (s, 1H), 7.61 – 7.51 (m, 2H), 7.48 (td, *J* = 8.7, 4.0 Hz, 1H), 7.44 – 7.33 (m, 2H), 6.49 (d, *J* = 3.6 Hz, 1H), 4.46 – 4.35 (m, 1H), 2.96 (s, 1H), 2.52 (s, 2H), 2.15 (s, 3H), 2.13 (dd, *J* = 30.7, 18.5 Hz, 2H), 2.02 (dd, *J* = 13.6, 6.8 Hz, 1H), 1.90 (s, 3H), 1.85 (dd, *J* = 13.6, 7.1 Hz, 1H), 1.79 – 1.55 (m, 4H).
LC/MS (APSI) m/z [M+H] calculated for C21H24F3N2O3: 409.2; found: 409.4.

rel-(1R,5R)-5-(2,5-dimethyl-1-phenyl-1H-pyrrole-3-carboxamido)-4,4-difluorocycloheptane-1-carboxylic acid – 83, Z4989072932, Z4989072932 **(Method 2).**
Yield: 50%; purity, >95, mixture of isomers% (assessed by LC/MS).
^{1}H NMR (600 MHz, DMSO-d_6) δ 12.19 (s, 1H), 7.53 (t, *J* = 7.6 Hz, 2H), 7.48 (d, *J* = 8.2 Hz, 2H), 7.29 – 7.22 (m, 2H), 6.45 (s, 1H), 4.41 (d, *J* = 19.0 Hz, 1H), 2.17 (s, 4H), 2.04 (dt, *J* = 14.3, 6.7 Hz, 1H), 1.91 (s, 4H), 1.86 (s, 1H), 1.78 – 1.56 (m, 3H), 1.66 (s, 2H).
LC/MS (APSI) m/z [M+H] calculated for C21H25F2N2O3: 391.2; found: 391.2.

4-(1-(4-fluorophenyl)-2,5-dimethyl-1H-pyrrole-3-carboxamido)cycloheptane-1-carboxylic acid – 119, 1757_0041, Z4906031088 **(Method 2).**
Yield: 43%; purity, >95%, mixture of isomers (assessed by LC/MS).
^{1}H NMR (400 MHz, DMSO-d_6) δ 7.25 (d, *J* = 6.6 Hz, 5H), 7.03 (d, *J* = 8.2 Hz, 1H), 6.27 (s, 1H), 3.92 – 3.85 (m, 1H), 2.54 (d, *J* = 1.4 Hz, 0H), 2.41 (d, *J* = 8.7 Hz, 2H), 2.20 (s, 4H), 1.96 (s, 4H), 2.05 – 1.81 (m, 4H), 1.80 – 1.61 (m, 1H), 1.61 – 1.43 (m, 2H).
LC/MS (APSI) m/z [M+H] calculated for C21H26FN2O3: 373.2; found: 373.4.

4-(1-(2,3-dihydrobenzo[b][1,4]dioxin-6-yl)-2,5-dimethyl-1H-pyrrole-3-carboxamido)cycloheptane-1-carboxylic acid – 81, 1757_0063, Z4909941677 **(Method 2).**
Yield: 41%; purity, >95% (assessed by LC/MS).
^{1}H NMR (600 MHz, DMSO-d_6) δ 11.97 (s, 1H), 7.26 (d, *J* = 8.1 Hz, 1H), 6.96 (d, *J* = 8.4 Hz, 1H), 6.76 (d, *J* = 2.4 Hz, 1H), 6.67 (dd, *J* = 8.5, 2.5 Hz, 1H), 6.31 (s, 1H), 4.28 (s, 4H), 3.84 (d, *J* = 10.3 Hz, 1H), 2.98 – 2.94 (m, 1H), 2.41 (s, 1H), 2.16 (s, 3H), 1.90 (s, 3H), 1.80 (dddt, *J* = 37.7, 16.9, 12.2, 5.8 Hz, 3H), 1.70 – 1.57 (m, 2H), 1.49 (dt, *J* = 13.8, 10.4 Hz, 4H).
LC/MS (APSI) m/z [M+H] calculated for C23H29N2O5: 413.2; found: 413.3.

4-(1-(3-fluorophenyl)-2,5-dimethyl-1H-pyrrole-3-carboxamido)cycloheptane-1-carboxylic acid – 126, 1757_0040, Z4906031087 **(Method 2).**
Yield: 36%; purity, >95%, mixture of isomers (assessed by LC/MS).
^{1}H NMR (600 MHz, DMSO-d_6) δ 11.97 (s, 1H), 7.56 (q, *J* = 7.7 Hz, 1H), 7.33 (dq, *J* = 8.5, 4.0, 3.4 Hz, 2H), 7.27 – 7.21 (m, 1H), 7.14 – 7.09 (m, 1H), 6.37 (s, 1H), 3.85 (s, 1H), 2.19 (s, 3H), 1.93 (s, 4H), 1.87 (q, *J* = 5.9, 4.9 Hz, 0H), 1.79 (ddd, *J* = 24.6, 15.5, 9.5 Hz, 2H), 1.71 – 1.58 (m, 2H), 1.49 (ddd, *J* = 16.3, 11.2, 6.3 Hz, 5H).
LC/MS (APSI) m/z [M+H] calculated for C21H26FN2O3: 373.2; found: 373.0.

4-(2,5-dimethyl-1-phenyl-1H-pyrrole-3-carboxamido)cycloheptane-1-carboxylic acid – 78, 4899_525, Z4658412025 **(Method 2).**
Yield: 27%; purity, >95% (assessed by LC/MS).
^{1}H NMR (400 MHz, DMSO-d_6) δ 11.71 (s, 1H), 7.55 – 7.40 (m, 3H), 7.24 – 7.17 (m, 2H), 7.00 (d, *J* = 8.1 Hz, 1H), 6.27 (s, 1H), 3.89 (s, 1H), 2.41 (d, *J* = 8.1 Hz, 1H), 2.21 (s, 3H), 1.97 (s, 3H), 1.94 (s, 1H), 1.85 (d, *J* = 10.2 Hz, 2H), 1.67 (s, 1H), 1.57 (s, 3H), 1.58 – 1.44 (m, 1H).
LC/MS (APSI) m/z [M+H] calculated for C21H27N2O3: 355.2; found: 355.2.

4-(1-(2-fluorophenyl)-2,5-dimethyl-1H-pyrrole-3-carboxamido)cycloheptane-1-carboxylic acid – 129, 1757_0039, Z4906031086 **(Method 2).**
Yield: 22%; purity, >95%, mixture of isomers (assessed by LC/MS).
^{1}H NMR (500 MHz, DMSO-d_6) δ 7.59 – 7.51 (m, 1H), 7.46 (t, *J* = 9.2 Hz, 1H), 7.43 – 7.29 (m, 3H), 6.41 (s, 1H), 3.81 (s, 1H), 2.14 (s, 3H), 2.09 (s, 1H), 1.88 (s, 3H), 1.80 (ddt, *J* = 16.7, 7.2, 4.2 Hz, 2H), 1.75 – 1.67 (m, 3H), 1.50 (s, 3H), 1.66 – 1.32 (m, 4H).
LC/MS (APSI) m/z [M+H] calculated for C21H26FN2O3: 373.2; found: 373.2.

2-((6-methylthieno[2,3-d]pyrimidin-4-yl)amino)spiro[3.5]nonane-7-carboxylic acid – 88, 4947_199, Z3464746067 **(Method 5).**
Yield: 39%; purity, >95% (assessed by LC/MS).
^{1}H NMR (600 MHz, DMSO-d_6) δ 11.99 (s, 1H), 8.22 (s, 1H), 7.79 (d, *J* = 7.0 Hz, 1H), 7.29 (d, *J* = 1.6 Hz, 1H), 2.51 (s, 1H), 2.26 (ddd, *J* = 11.7, 8.1, 4.0 Hz, 1H), 2.13 (ddd, *J* = 11.3, 7.8, 3.8 Hz, 2H), 1.72 (dddd, *J* = 40.7, 16.8, 11.7, 7.4 Hz, 5H), 1.64 – 1.58 (m, 1H), 1.47 – 1.28 (m, 4H).
LC/MS (APSI) m/z [M+H] calculated for C17H22N3O2S: 332.2; found: 332.1.

2-((7-methyl-5,6,7,8-tetrahydrobenzo[4,5]thieno[2,3-d]pyrimidin-4-yl)amino)spiro[3.5]nonane-7-carboxylic acid – 92, 4947_245, Z4647738795 **(Method 5).**
Yield: 19%; purity, >95% (assessed by LC/MS).
^{1}H NMR (600 MHz, DMSO-d_6) δ 8.23 (s, 1H), 6.31 (d, *J* = 7.0 Hz, 1H), 4.54 (q, *J* = 8.0 Hz, 1H), 3.07 (d, *J* = 16.2 Hz, 1H), 2.84 (d, *J* = 14.9 Hz, 1H), 2.24 (s, 1H), 2.12 (d, *J* = 11.5 Hz, 2H), 1.91 (s, 1H), 1.84 (t, *J* = 9.3 Hz, 0H), 1.79 – 1.70 (m, 2H), 1.66 (d, *J* = 9.2 Hz, 1H), 1.48 – 1.40 (m, 1H), 1.34 (s, 2H), 1.05 (d, *J* = 6.5 Hz, 2H).
LC/MS (APSI) m/z [M+H] calculated for C21H28N3O2S: 386.2; found: 386.2.

N-(3-(1H-tetrazol-5-yl)phenyl)-6-ethyl-2-methylnicotinamide – 63, ZINC000354919126, Z1680371362 **(Method 1).**
Yield: 28%; purity, >95% (assessed by LC/MS).
^{1}H NMR (600 MHz, dmso) δ 10.61, 8.55, 7.82, 7.81, 7.80, 7.74, 7.73, 7.57, 7.55, 7.54, 7.22, 7.21, 3.29, 2.77, 2.76, 2.75, 2.73, 2.55, 2.49, 2.49, 2.48, 2.48, 2.48, 1.24, 1.23, 1.21.
LC/MS (APSI) m/z [M+H] calculated for C16H17N6O: 309.2; found: 309.2.

3-(ethylthio)-4-(1-(2-fluorophenyl)-1H-pyrazole-4-carboxamido)benzoic acid – Z4361714909, Z4361714909 (Method 2).
Yield: 25%; purity, >95% (assessed by LC/MS).
^{1}H NMR (600 MHz, DMSO-d_6) δ 13.05 (s, 1H), 9.82 (s, 1H), 8.86 (d, *J* = 2.1 Hz, 1H), 8.32 (s, 1H), 7.96 (t, *J* = 2.0 Hz, 1H), 7.88 – 7.78 (m, 2H), 7.69 (t, *J* = 8.7 Hz, 1H), 7.55 – 7.46 (m, 2H), 7.39 (td, *J* = 7.5, 7.1, 1.9 Hz, 1H), 2.94 (q, *J* = 7.3 Hz, 2H), 1.20 (t, *J* = 7.3 Hz, 3H).
LC/MS (APSI) m/z [M+H] calculated for C19H17FN3O3S: 386.1; found: 386.0.
1-(4-chlorophenyl)-N,5-dimethyl-N-(1H-tetrazol-5-yl)-1H-pyrazole-4-carboxamide – 69, ZINC001131170402, Z4175999422 **(Method 1).**
Yield: 47%; purity, >95% (assessed by LC/MS).
^{1}H NMR (600 MHz, DMSO-d_6) δ 11.26 (s, 1H), 8.37 (s, 1H), 7.66 – 7.54 (m, 5H), 3.91 (s, 2H), 2.53 (s, 2H).
LC/MS (APSI) m/z [M+H] calculated for C13H13ClN7O: 318.1; found: 318.0.

2-(methyl(9H-pyrimido[4,5-b]indol-4-yl)amino)spiro[3.5]nonane-7-carboxylic acid – 76, SG-0002, Z4877468173 **(Method 5).**
Yield: 55%; purity, >95% (assessed by LC/MS).
^{1}H NMR (600 MHz, DMSO-d_6) δ 8.38 (s, 1H), 7.83 (d, *J* = 8.0 Hz, 1H), 7.48 (d, *J* = 8.0 Hz, 1H), 7.39 (t, *J* = 7.6 Hz, 1H), 7.27 (t, *J* = 7.6 Hz, 1H), 4.64 (p, *J* = 8.5 Hz, 1H), 3.14 (s, 3H), 2.22 (ddd, *J* = 11.7, 8.0, 4.0 Hz, 1H), 2.09 (ddd, *J* = 11.8, 8.0, 4.0 Hz, 2H), 2.00 (dd, *J* = 11.3, 8.7 Hz, 1H), 1.92 (dd, *J* = 11.4, 8.8 Hz, 1H), 1.71 (d, *J* = 10.9 Hz, 2H), 1.65 (d, *J* = 11.9 Hz, 1H).
LC/MS (APSI) m/z [M+H] calculated for C21H25N4O2: 365.2; found: 365.2.

2-(3-(5-bromo-1-methyl-2-oxo-1,2-dihydro-3H-pyrrolo [2,3-b] pyridin-3-ylidene) methyl)phenoxy)acetic acid – 62, ZINC000373547885, Z1956737209 **(Method 4).**
Yield: 44%; purity, >95, mixture of isomers% (assessed by LC/MS).
^{1}H NMR (500 MHz, DMSO-d_6) δ 7.89 (s, 1H), 7.85 (q, *J* = 3.0 Hz, 1H), 7.48 (t, *J* = 8.0 Hz, 1H), 7.42 (t, *J* = 8.0 Hz, 1H), 7.32 (d, *J* = 7.6 Hz, 1H), 7.25 (d, *J* = 2.6 Hz, 1H), 7.08 (dt, *J* = 7.8, 3.2 Hz, 1H), 4.72 (d, *J* = 4.2 Hz, 2H), 3.20 (d, *J* = 5.0 Hz, 3H).
LC/MS (APSI) m/z [M+H] calculated for C17H14BrN2O4: 389.0; found: 389.0.

2-((9-methyl-9H-pyrimido[4,5-b]indol-4-yl)amino)spiro[3.5]nonane-7-carboxylic acid – 75, 7827_0009, Z4875089792 **(Method 3).**
Yield: 34%; purity, >95% (assessed by LC/MS).
^{1}H NMR (600 MHz, DMSO-d_6) δ 8.42 (d, *J* = 7.8 Hz, 1H), 8.37 (s, 1H), 7.59 (d, *J* = 8.1 Hz, 1H), 7.44 (t, *J* = 7.7 Hz, 1H), 7.29 (t, *J* = 7.5 Hz, 1H), 3.81 (s, 3H), 2.14 (tt, *J* = 10.3, 4.6 Hz, 2H), 2.06 – 1.96 (m, 1H), 1.93 (t, *J* = 9.9 Hz, 1H), 1.80 (d, *J* = 13.0 Hz, 1H), 1.68 (q, *J* = 8.1, 6.2 Hz, 2H), 1.42 – 1.36 (m, 2H), 1.34 (d, *J* = 11.3 Hz, 1H).
LC/MS (APSI) m/z [M+H] calculated for C21H25N4O2: 365.2; found: 365.2.

benzyl 3-((1-methyl-1H-tetrazol-5-yl)amino)-2-phenylpropanoate – 67, ZINC000786823916, Z1815900911 **(Method 3).**
Yield: 23%; purity, >95% (assessed by LC/MS).
^{1}H NMR (500 MHz, Acetonitrile-d_3) δ 7.41 – 7.28 (m, 7H), 7.26 (dt, *J* = 8.0, 2.9 Hz, 2H), 5.38 (d, *J* = 6.1 Hz, 1H), 5.14 (d, *J* = 2.3 Hz, 2H), 4.19 (ddd, *J* = 9.2, 6.3, 2.7 Hz, 1H), 4.02 (dddd, *J* = 11.6, 9.0, 6.3, 2.7 Hz, 1H), 3.68 (dtd, *J* = 15.0, 6.2, 2.4 Hz, 1H), 3.59 (d, *J* = 2.6 Hz, 3H).
LC/MS (APSI) m/z [M+H] calculated for C18H20N5O2: 338.2; found: 338.0.

4-(1-(4-methoxyphenyl)-2,5-dimethyl-1H-pyrrole-3-carboxamido)cycloheptane-1-carboxylic acid – 124, 1757_0077, Z4909941691 **(Method 2).**
Yield: 52%; purity, >95% (assessed by LC/MS).
^{1}H NMR (600 MHz, DMSO-d_6) δ 11.97 (s, 1H), 7.27 (d, *J* = 8.1 Hz, 1H), 7.17 – 7.12 (m, 2H), 7.07 – 7.01 (m, 2H), 6.33 (s, 1H), 3.80 (s, 3H), 2.97 (s, 1H), 2.41 (s, 1H), 2.15 (s, 3H), 1.89 (s, 4H), 1.88 – 1.78 (m, 2H), 1.76 (d, *J* = 13.6 Hz, 1H), 1.71 – 1.57 (m, 2H), 1.55 – 1.43 (m, 4H).
LC/MS (APSI) m/z [M+H] calculated for C22H29N2O4: 385.2; found: 385.2.

4-(1-(3-methoxyphenyl)-2,5-dimethyl-1H-pyrrole-3-carboxamido)cycloheptane-1-carboxylic acid – 117, 1757_0078, Z4909941692 **(Method 2).**
Yield: 59%; purity, >95% (assessed by LC/MS).
^{1}H NMR (600 MHz, DMSO-d_6) δ 8.49 (s, 0H), 7.42 (t, *J* = 8.3 Hz, 1H), 7.29 (d, *J* = 8.1 Hz, 1H), 7.03 (dd, *J* = 8.4, 2.5 Hz, 1H), 6.79 (dd, *J* = 4.5, 2.2 Hz, 2H), 6.35 (s, 1H), 3.84 (s, 1H), 3.78 (s, 3H), 2.37 (s, 1H), 2.18 (s, 3H), 1.92 (s, 3H), 1.91 – 1.72 (m, 3H), 1.70 – 1.57 (m, 2H), 1.55 – 1.42 (m, 4H).
LC/MS (APSI) m/z [M+H] calculated for C22H29N2O4: 385.2; found: 385.4.

N- (3- (1H-tetrazol-5-yl) phenyl) -3- (4-cyanophenoxy) methyl) benzamide – 70, Z1211720932, Z1211720932 **(Method 1).**
Yield: 23%; purity, >95% (assessed by LC/MS).
^{1}H NMR (500 MHz, DMSO-d_6) δ 10.55 (s, 1H), 8.57 (s, 1H), 8.07 (s, 1H), 7.96 (dd, *J* = 17.2, 8.0 Hz, 2H), 7.78 (d, *J* = 8.3 Hz, 2H), 7.74 (d, *J* = 7.8 Hz, 1H), 7.68 (d, *J* = 7.6 Hz, 1H), 7.57 (q, *J* = 7.1 Hz, 2H), 7.21 (d, *J* = 8.4 Hz, 2H), 5.30 (s, 2H).
LC/MS (APSI) m/z [M+H] calculated for C22H17N6O2: 397.1; found: 397.2.

rel-(1R,5R)-4,4-difluoro-5-(5-phenyl-1H-pyrrole-3-carboxamido)cycloheptane-1-carboxylic acid – 80, 1757_0002, Z4906033301 **(Method 2).**
Yield: 46%; purity, 94% (assessed by LC/MS).
^{1}H NMR (600 MHz, DMSO-d_6) δ 12.21 (s, 1H), 11.67 (s, 1H), 7.75 (d, *J* = 9.1 Hz, 1H), 7.67 – 7.58 (m, 3H), 7.49 (d, *J* = 1.9 Hz, 1H), 7.36 (t, *J* = 7.6 Hz, 3H), 7.19 (q, *J* = 8.7, 7.3 Hz, 2H), 7.02 – 6.95 (m, 1H), 2.15 (dd, *J* = 13.7, 7.2 Hz, 1H), 2.05 (d, *J* = 9.6 Hz, 1H), 1.92 – 1.84 (m, 1H), 1.81 – 1.60 (m, 3H).
LC/MS (APSI) m/z [M+H] calculated for C19H21F2N2O3: 363.2; found: 363.2.

rel-(1R,5R)-5-(5-cyclohexylthiophene-3-carboxamido)-4,4-difluorocycloheptane-1-carboxylic acid – 110, 4899_1757, Z4658412048 **(Method 2).**
Yield: 45%; purity, >95% (assessed by LC/MS).
^{1}H NMR (600 MHz, DMSO-d_6) δ 12.22 (s, 1H), 8.19 (d, *J* = 9.2 Hz, 1H), 7.97 (d, *J* = 1.4 Hz, 1H), 7.28 (s, 1H), 4.39 (dd, *J* = 20.3, 11.2 Hz, 1H), 2.81 – 2.73 (m, 1H), 2.16 (tt, *J* = 14.9, 7.9 Hz, 1H), 2.14 (s, 1H), 2.15 – 2.00 (m, 1H), 1.96 (dd, *J* = 8.5, 4.2 Hz, 2H), 1.91 – 1.77 (m, 1H), 1.81 – 1.55 (m, 6H), 1.42 – 1.28 (m, 5H), 1.25 – 1.14 (m, 1H).
LC/MS (APSI) m/z [M+H] calculated for C19H26F2NO3S: 386.2; found: 386.2.

2-(5-cyclohexylthiophene-3-carboxamido)spiro[3.5]nonane-7-carboxylic acid – 109, 4899_1507, Z4658412001 **(Method 2).**
Yield: 31%; purity, >95% (assessed by LC/MS).
^{1}H NMR (600 MHz, DMSO-d_6) δ 12.01 (s, 1H), 8.23 (d, *J* = 7.6 Hz, 1H), 7.84 (d, *J* = 1.4 Hz, 1H), 7.22 (s, 1H), 4.28 (p, *J* = 8.2 Hz, 1H), 2.75 (tt, *J* = 8.1, 3.6 Hz, 1H), 2.13 (dddt, *J* = 17.7, 10.7, 6.8, 3.8 Hz, 2H), 2.01 (ddd, *J* = 11.8, 8.2, 4.0 Hz, 1H), 1.95 (d, *J* = 8.0 Hz, 2H), 1.78 – 1.72 (m, 2H), 1.75 – 1.62 (m, 5H), 1.59 (d, *J* = 13.3 Hz, 1H), 1.45 – 1.26 (m, 9H), 1.19 (ddq, *J* = 12.7, 9.1, 5.3, 4.5 Hz, 1H).
LC/MS (APSI) m/z [M+H] calculated for C21H30NO3S: 376.2; found: 376.2.

2-((2-(trifluoromethyl)quinazolin-4-yl)amino)spiro[3.5]nonane-7-carboxylic acid – 90, 4947_7809, Z4647716414 **(Method 5).**
Yield: 21%; purity, >95% (assessed by LC/MS).
^{1}H NMR (600 MHz, DMSO-d_6) δ 11.97 (s, 1H), 8.78 (d, *J* = 6.4 Hz, 1H), 8.42 (d, *J* = 8.2 Hz, 1H), 7.86 (td, *J* = 7.5, 6.8, 1.3 Hz, 1H), 7.80 (d, *J* = 8.1 Hz, 1H), 7.69 – 7.63 (m, 1H), 4.61 (td, *J* = 8.4, 6.5 Hz, 1H), 2.31 (ddd, *J* = 11.9, 8.2, 3.9 Hz, 1H), 1.92 (dd, *J* = 11.4, 8.2 Hz, 1H), 1.87 (dd, *J* = 11.4, 8.2 Hz, 1H), 1.82 – 1.72 (m, 2H), 1.71 – 1.62 (m, 2H), 1.48 – 1.29 (m, 4H).
LC/MS (APSI) m/z [M+H] calculated for C19H21F3N3O2: 380.2; found: 380.2.

2-(benzofuro[3,2-d]pyrimidin-4-ylamino)spiro[3.5]nonane-7-carboxylic acid – 84, 4947_7855, Z4647716483 **(Method 5).**
Yield: 23%; purity, >95% (assessed by LC/MS).
^{1}H NMR (600 MHz, DMSO-d_6) δ 8.44 (s, 1H), 8.30 (d, *J* = 7.2 Hz, 1H), 8.05 (d, *J* = 7.7 Hz, 1H), 7.77 (d, *J* = 8.4 Hz, 1H), 7.67 (t, *J* = 7.8 Hz, 1H), 7.47 (t, *J* = 7.5 Hz, 1H), 2.27 (s, 1H), 2.17 – 2.10 (m, 2H), 1.90 (dd, *J* = 11.3, 8.2 Hz, 1H), 1.65 (t, *J* = 13.5 Hz, 2H), 1.43 (q, *J* = 11.9 Hz, 1H), 1.34 (dq, *J* = 23.1, 10.6 Hz, 3H).
LC/MS (APSI) m/z [M+H] calculated for C20H22N3O3: 352.2; found: 352.2.

2-(8-fluoro-9H-pyrimido [4,5-b] indol-4-yl) amino) spiro [3.5] non-ane-7-carboxylic acid – 74 7827_0016, Z4875089799 **(Method 5).**
Yield: 53%; purity, >95% (assessed by LC/MS).
^{1}H NMR (400 MHz, DMSO-d_6) δ 12.38 (s, 1H), 12.07 (s, 1H), 8.36 (s, 1H), 8.27 – 8.19 (m, 1H), 7.29 – 7.19 (m, 2H), 7.15 (d, *J* = 7.4 Hz, 1H), 2.54 (s, 1H), 2.29 (td, *J* = 10.2, 8.3, 4.4 Hz, 1H), 2.16 (s, 2H), 1.79 (t, *J* = 15.5 Hz, 2H), 1.71 (d, *J* = 8.5 Hz, 2H), 1.54 – 1.38 (m, 1H), 1.38 (s, 2H).
LC/MS (APSI) m/z [M+H] calculated for C20H22FN4O2: 369.2; found: 369.2.

2-((7-fluoro-9H-pyrimido[4,5-b]indol-4-yl)amino)spiro[3.5]nonane-7-carboxylic acid – 134, 7827_0015, Z4875089798 **(Method 5).**
Yield: 43%; purity, >95% (assessed by LC/MS).
^{1}H NMR (400 MHz, DMSO-d_6) δ 11.77 (s, 1H), 8.29 (dd, *J* = 8.7, 5.3 Hz, 1H), 8.19 (s, 1H), 7.12 (dd, *J* = 9.5, 2.4 Hz, 1H), 6.95 (td, *J* = 9.1, 2.5 Hz, 1H), 6.82 (d, *J* = 7.4 Hz, 1H), 4.81 (p, *J* = 8.1 Hz, 1H), 2.35 (s, 1H), 2.18 (dd, *J* = 25.8, 14.1 Hz, 3H), 2.06 – 1.94 (m, 1H), 1.93 (s, 2H), 1.82 (d, *J* = 13.3 Hz, 1H), 1.75 (d, *J* = 9.1 Hz, 2H).
LC/MS (APSI) m/z [M+H] calculated for C20H22FN4O2: 369.2; found: 369.2.

2-((6-bromo-9H-pyrimido [4,5-b] indol-4-yl) amino) spiro [3.5] non-ane-7-carboxylic acid – 135, 7827_0018, Z4875089801 **(Method 5).**
Yield: 33%; purity, >95% (assessed by LC/MS).
^{1}H NMR (400 MHz, DMSO-d_6) δ 12.05 (s, 1H), 8.65 (s, 1H), 8.34 (s, 1H), 7.49 (d, *J* = 8.6 Hz, 1H), 7.39 (d, *J* = 8.4 Hz, 1H), 7.27 (d, *J* = 7.2 Hz, 1H), 4.85 – 4.76 (m, 1H), 2.30 (s, 1H), 2.16 (s, 2H), 1.97 (dt, *J* = 29.2, 9.9 Hz, 2H), 1.80 (t, *J* = 15.6 Hz, 2H), 1.71 (d, *J* = 9.3 Hz, 2H), 1.44 (dd, *J* = 30.6, 11.9 Hz, 4H).
LC/MS (APSI) m/z [M+H] calculated for C20H22BrN4O2: 431.1; found: 431.0.

2-((9H-pyrimido[4,5-b]indol-4-yl)amino)spiro[3.5]nonane-7-carboxylic acid – 73, 4947num7827, Z4647716459 **(Method 5).**
Yield: 34%; purity, >95% (assessed by LC/MS).
^{1}H NMR (400 MHz, dmso) δ 11.86, 8.40, 8.38, 8.32, 7.45, 7.43, 7.38, 7.36, 7.34, 7.26, 7.24, 7.23, 7.05, 7.03, 4.84, 4.82, 4.80, 3.36, 3.15, 2.50, 2.32, 2.29, 2.16, 2.07, 2.03, 2.01, 1.98, 1.96, 1.94, 1.91, 1.83, 1.79, 1.75, 1.70, 1.70, 1.49, 1.46, 1.40, 1.37, 0.94, 0.93.
LC/MS (APSI) m/z [M-H] calculated for C20H21N4O2: 349.2; found: 349.2.

2-((9H-pyrido[3',2':4,5]pyrrolo[2,3-d]pyrimidin-4-yl)amino)spiro[3.5]nonane-7-carboxylic acid – 133, 7827_0004, Z4875089787 **(Method 5).**
Yield: 30%; purity, >95% (assessed by LC/MS).
^{1}H NMR (400 MHz, DMSO-d_6) δ 12.39 (s, 1H), 12.04 (s, 1H), 8.79 (d, *J* = 7.8 Hz, 1H), 8.37 (d, *J* = 5.1 Hz, 2H), 7.33 – 7.20 (m, 2H), 2.54 (s, 1H), 2.28 (d, *J* = 9.8 Hz, 1H), 2.22 – 2.11 (m, 2H), 1.96 (dt, *J* = 29.0, 9.9 Hz, 2H), 1.86 – 1.66 (m, 4H), 1.54 – 1.32 (m, 4H).
LC/MS (APSI) m/z [M+H] calculated for C19H22N5O2: 352.2; found: 352.2.

2-((6-phenylthieno[2,3-d]pyrimidin-4-yl)amino)spiro[3.5]nonane-7-carboxylic acid – 85, 4947_7823, Z4647716437 **(Method 5).**
Yield: 22%; purity, >95% (assessed by LC/MS).
^{1}H NMR (600 MHz, DMSO-d_6) δ 12.00 (s, 1H), 8.30 (s, 1H), 8.03 (d, *J* = 16.1 Hz, 1H), 7.67 (d, *J* = 7.6 Hz, 2H), 7.49 (t, *J* = 7.6 Hz, 2H), 7.38 (t, *J* = 7.4 Hz, 1H), 4.60 (td, *J* = 8.6, 5.6 Hz, 1H), 2.31 (t, *J* = 8.2 Hz, 1H), 2.22 – 2.12 (m, 2H), 1.71 – 1.66 (m, 1H), 1.63 (d, *J* = 12.3 Hz, 1H), 1.44 (d, *J* = 13.4 Hz, 1H), 1.42 – 1.32 (m, 3H).
LC/MS (APSI) m/z [M+H] calculated for C22H24N3O2S: 394.2; found: 394.2.

2-(benzo[4,5]thieno[2,3-d]pyrimidin-4-ylamino)spiro[3.5]nonane-7-carboxylic acid – 72, 4947num7838, Z4647716479 **(Method 5).**
Yield: 17%; purity, >95% (assessed by LC/MS).
^{1}H NMR (600 MHz, DMSO-d_6) δ 8.46 (d, *J* = 13.0 Hz, 2H), 8.06 (d, *J* = 7.9 Hz, 1H), 7.58 (t, *J* = 7.6 Hz, 1H), 7.52 (t, *J* = 7.6 Hz, 1H), 7.31 (d, *J* = 6.9 Hz, 1H), 2.30 (ddd, *J* = 11.6, 8.1, 4.1 Hz, 1H), 2.16 (tdd, *J* = 11.7, 7.6, 3.9 Hz, 2H), 2.07 – 1.98 (m, 1H), 1.94 (dd, *J* = 11.1, 8.5 Hz, 1H), 1.81 (dd, *J* = 10.6, 6.4 Hz, 1H), 1.74 – 1.65 (m, 2H), 1.42 – 1.29 (m, 3H).
LC/MS (APSI) m/z [M+H] calculated for C20H22N3O2S: 368.2; found: 368.1.

2-((6,7-dihydro-5H-cyclopenta[4,5]thieno[2,3-d]pyrimidin-4-yl)amino)spiro[3.5]nonane-7-carboxylic acid – 91, 4947_7831, Z4647716465 **(Method 5).**
Yield: 23%; purity, >95% (assessed by LC/MS).
^{1}H NMR (500 MHz, DMSO-d_6) δ 8.23 (s, 1H), 6.44 (d, *J* = 7.1 Hz, 1H), 4.56 (h, *J* = 8.0 Hz, 1H), 3.10 (t, *J* = 7.0 Hz, 2H), 2.91 (t, *J* = 7.3 Hz, 2H), 2.40 (q, *J* = 7.4 Hz, 1H), 2.39 (s, 1H), 2.23 (ddd, *J* = 11.6, 8.1, 4.1 Hz, 1H), 2.10 (ddd, *J* = 11.9, 8.5, 4.5 Hz, 2H), 1.75 (s, 1H), 1.65 (d, *J* = 9.6 Hz, 2H), 1.38 – 1.29 (m, 4H).
LC/MS (APSI) m/z [M+H] calculated for C19H24N3O2S: 358.2; found: 358.2.

2-((5,6,7,8-tetrahydrobenzo[4,5]thieno[2,3-d]pyrimidin-4-yl)amino)spiro[3.5]nonane-7-carboxylic acid – 71, Z4361714947, Z4361714947 **(Method 5).**
Yield: 70%; purity, >95% (assessed by LC/MS).
^{1}H NMR (500 MHz, DMSO-d_6) δ 8.44 (s, 1H), 8.23 (s, 1H), 4.54 (q, *J* = 7.9 Hz, 1H), 2.98 (d, *J* = 5.1 Hz, 2H), 2.74 (d, *J* = 5.2 Hz, 2H), 2.23 (ddd, *J* = 11.6, 8.0, 4.1 Hz, 1H), 2.10 (ddt, *J* = 12.3, 8.7, 4.2 Hz, 2H), 1.86 – 1.67 (m, 7H), 1.70 – 1.60 (m, 2H), 1.41 (q, *J* = 10.6 Hz, 1H), 1.38 – 1.23 (m, 3H).
LC/MS (APSI) m/z [M+H] calculated for C20H26N3O2S: 372.2; found: 372.2.

2-((5-phenylpyrazolo[1,5-a]pyrimidin-7-yl)amino)spiro[3.5]nonane-7-carboxylic acid – 89, 4947_7885, Z4647716494 **(Method 5).**
Yield: 17%; purity, >95% (assessed by LC/MS).
^{1}H NMR (600 MHz, DMSO-d_6) δ 11.99 (s, 1H), 8.15 (d, *J* = 7.4 Hz, 2H), 8.08 (d, *J* = 2.2 Hz, 1H), 8.04 (d, *J* = 7.3 Hz, 1H), 7.47 (dd, *J* = 11.5, 7.0 Hz, 3H), 6.58 (s, 1H), 6.45 (d, *J* = 2.2 Hz, 1H), 4.39 (p, *J* = 7.8 Hz, 1H), 2.27 – 2.20 (m, 1H), 2.07 – 1.99 (m, 1H), 1.91 (dd, *J* = 11.5, 7.9 Hz, 2H), 1.79 – 1.72 (m, 1H), 1.67 (q, *J* = 10.4, 8.8 Hz, 2H), 1.35 (dq, *J* = 20.2, 11.7 Hz, 3H).
LC/MS (APSI) m/z [M+H] calculated for C22H25N4O2: 377.2; found: 377.2.

4-(1-cyclohexyl-1H-pyrazole-4-carboxamido)cycloheptane-1-carboxylic acid – 112, 4899_678, Z3509940601 **(Method 5).**
Yield: 33%; purity, >95% (assessed by LC/MS).
^{1}H NMR (500 MHz, DMSO-d_6) δ 12.01 (s, 1H), 8.16 (s, 1H), 7.80 (s, 1H), 7.74 (d, *J* = 7.9 Hz, 1H), 4.10 (tt, *J* = 11.5, 3.9 Hz, 1H), 3.84 (tt, *J* = 8.5, 4.5 Hz, 1H), 2.40 (d, *J* = 6.7 Hz, 1H), 2.02 – 1.95 (m, 2H), 1.87 (qt, *J* = 12.2, 5.7 Hz, 2H), 1.83 – 1.73 (m, 4H), 1.64 (qd, *J* = 13.0, 12.0, 5.0 Hz, 5H), 1.49 (s, 1H), 1.58 – 1.30 (m, 5H), 1.18 (dddd, *J* = 16.7, 13.0, 8.3, 3.7 Hz, 1H).
LC/MS (APSI) m/z [M+H] calculated for C18H28N3O3: 334.2; found: 334.2.

4-(5,6-dihydro-4H-cyclopenta[b]thiophene-2-carboxamido)cycloheptane-1-carboxylic acid – 104, 4899_533**,** Z3375989610 **(Method 2).**
Yield: 50%; purity, >95% (assessed by LC/MS).
^{1}H NMR (600 MHz, DMSO-d_6) δ 8.47 (s, 1H), 7.53 (s, 1H), 3.82 (s, 1H), 3.42 – 3.38 (m, 2H), 2.83 (t, *J* = 7.3 Hz, 2H), 2.66 (t, *J* = 7.3 Hz, 2H), 2.52 (s, 2H), 2.35 (p, *J* = 7.4 Hz, 3H), 1.86 (dt, *J* = 12.3, 4.0 Hz, 2H), 1.86 – 1.73 (m, 2H), 1.69 – 1.56 (m, 1H), 1.49 (dp, *J* = 20.7, 10.5, 9.8 Hz, 3H).
LC/MS (APSI) m/z [M+H] calculated for C16H22NO3S: 308.2; found: 308.1.

4-(5-cyclohexylthiophene-3-carboxamido)cycloheptane-1-carboxylic acid – 66, Z4361714899, Z4361714899 **(Method 2).**
Yield: 58%; purity, >95% (assessed by LC/MS).
^{1}H NMR (500 MHz, DMSO-d_6) δ 7.93 (d, *J* = 8.0 Hz, 1H), 7.84 (s, 1H), 7.22 (s, 1H), 3.85 (dq, *J* = 9.7, 4.8 Hz, 1H), 2.75 (s, 1H), 2.49 (s, 1H), 2.43 – 2.36 (m, 1H), 1.95 (d, *J* = 7.8 Hz, 2H), 1.90 – 1.83 (m, 1H), 1.86 – 1.71 (m, 3H), 1.74 – 1.56 (m, 3H), 1.50 (tt, *J* = 12.5, 4.8 Hz, 4H), 1.35 (h, *J* = 10.6, 10.1 Hz, 4H), 1.20 (ddd, *J* = 16.3, 8.4, 3.8 Hz, 1H), 0.98 (s, 1H).
LC/MS (APSI) m/z [M+H] calculated for C19H28NO3S: 350.2; found: 350.1.

4-(5-benzylthiophene-3-carboxamido)cycloheptane-1-carboxylic acid – 107, 4899_455, Z4658412023 **(Method 2).**
Yield: 30%; purity, >95% (assessed by LC/MS).
^{1}H NMR (500 MHz, DMSO-d_6) δ 12.11 (s, 1H), 7.97 (d, *J* = 7.9 Hz, 1H), 7.88 (s, 1H), 7.30 (t, *J* = 7.4 Hz, 2H), 7.23 (dd, *J* = 14.3, 8.1 Hz, 4H), 4.10 (s, 2H), 3.82 (d, *J* = 10.5 Hz, 1H), 2.42 – 2.36 (m, 2H), 1.92 – 1.74 (m, 4H), 1.64 (dq, *J* = 22.3, 7.4, 7.0 Hz, 2H), 1.54 – 1.41 (m, 4H).
LC/MS (APSI) m/z [M+H] calculated for C20H24NO3S: 358.2; found: 358.0.

4-(1-(3-fluorophenyl)-1,4,5,6-tetrahydrocyclopenta[c]pyrazole-3-carboxamido)cycloheptane-1-carboxylic acid – 123, 1757_0055, Z4906031102 **(Method 2).**
Yield: 67%; purity, >95% (assessed by LC/MS).
^{1}H NMR (600 MHz, DMSO-d_6) δ 12.00 (s, 1H), 7.90 (d, *J* = 8.3 Hz, 1H), 7.71 – 7.66 (m, 1H), 7.54 (q, *J* = 8.3, 7.3 Hz, 3H), 7.17 (ddd, *J* = 10.6, 7.1, 2.8 Hz, 1H), 3.87 (dt, *J* = 9.6, 5.0 Hz, 1H), 3.05 (t, *J* = 7.1 Hz, 3H), 2.68 (t, *J* = 7.1 Hz, 3H), 2.55 (d, *J* = 7.0 Hz, 1H), 1.88 (td, *J* = 17.0, 15.3, 4.8 Hz, 2H), 1.80 (dt, *J* = 9.8, 4.8 Hz, 2H), 1.72 – 1.64 (m, 1H), 1.64 – 1.46 (m, 4H).
LC/MS (APSI) m/z [M+H] calculated for C21H25FN3O3: 386.2; found: 386.0.

4-(1-phenyl-1,4,5,6-tetrahydrocyclopenta[c]pyrazole-3-carboxamido)cycloheptane-1-carboxylic acid – 98, 4899_583, Z3509941223 **(Method 2).**
Yield: 40%; purity, >95% (assessed by LC/MS).
^{1}H NMR (600 MHz, DMSO-d_6) δ 12.00 (s, 1H), 7.80 (d, *J* = 8.4 Hz, 1H), 7.77 – 7.72 (m, 2H), 7.50 (t, *J* = 7.9 Hz, 2H), 7.33 (t, *J* = 7.4 Hz, 1H), 3.87 (tt, *J* = 9.0, 4.4 Hz, 1H), 3.01 (t, *J* = 7.2 Hz, 2H), 2.69 (t, *J* = 7.2 Hz, 2H), 2.54 (q, *J* = 7.3 Hz, 2H), 2.41 (ddd, *J* = 8.1, 6.1, 3.9 Hz, 1H), 1.88 (qt, *J* = 11.7, 5.3 Hz, 2H), 1.80 (ddd, *J* = 11.1, 8.3, 4.4 Hz, 2H), 1.72 – 1.52 (m, 3H), 1.54 – 1.46 (m, 1H).
LC/MS (APSI) m/z [M+H] calculated for C21H26N3O3: 368.2; found: 368.3.

2-(4-(2-cyclopropyl-6-fluoroquinoline-4-carboxamido)phenyl)acetic acid – 68, ZINC000431975755, Z1870131726 **(Method 2).**
Yield: 31%; purity, >95% (assessed by LC/MS).
^{1}H NMR (500 MHz, DMSO-d_6) δ 12.32 – 12.27 (m, 1H), 10.71 (s, 1H), 7.98 (dd, *J* = 9.2, 5.5 Hz, 1H), 7.77 (dd, *J* = 10.2, 3.0 Hz, 1H), 7.73 – 7.67 (m, 3H), 7.65 (td, *J* = 8.8, 2.8 Hz, 1H), 7.26 (d, *J* = 8.1 Hz, 2H), 3.55 (s, 2H), 2.41 – 2.33 (m, 1H), 1.18 – 1.05 (m, 4H).
LC/MS (APSI) m/z [M+H] calculated for C21H18FN2O3: 365.1; found: 365.2.

2-(1-((2'R,4S)-spiro[chromane-4,1'-cyclopropane]-2'-carbonyl)indolin-3-yl)acetic acid – 65, ZINC000739642212, Z2755710279 **(Method 2).**
Yield: 30%; purity, >95% (assessed by LC/MS).
^{1}H NMR (600 MHz, DMSO-d_6) δ 12.28 (s, 1H), 7.26 (d, *J* = 7.4 Hz, 1H), 7.16 (t, *J* = 7.8 Hz, 1H), 7.10 – 7.04 (m, 1H), 6.99 (t, *J* = 7.4 Hz, 1H), 6.94 (dd, *J* = 7.9, 1.7 Hz, 1H), 6.85 (t, *J* = 7.5 Hz, 1H), 6.74 (d, *J* = 8.1 Hz, 1H), 4.49 (t, *J* = 9.9 Hz, 1H), 4.18 (ddd, *J* = 10.3, 6.4, 3.2 Hz, 1H), 4.03 (ddd, *J* = 11.0, 8.2, 3.0 Hz, 1H), 3.63 (ddd, *J* = 32.2, 9.9, 5.1 Hz, 2H), 2.67 (dd, *J* = 16.6, 4.8 Hz, 1H), 2.51 (d, *J* = 6.2 Hz, 1H), 1.98 – 1.84 (m, 2H), 1.59 (t, *J* = 5.3 Hz, 1H), 1.47 (dd, *J* = 8.3, 4.7 Hz, 1H).
LC/MS (APSI) m/z [M+H] calculated for C22H22NO4: 364.2; found: 364.0.

2-((8-fluoro-9H-pyrimido[4,5-b]indol-4-yl)amino)spiro[3.5]nonane-7-carboxylic acid – 74, 77, 7827_0016, Z4875089799, Z5090949977 **(Method 5).**
Yield: 70%; purity, >95% (assessed by LC/MS).
^{1}H NMR (400 MHz, DMSO-d_6) δ 8.35 (s, 1H), 8.27 – 8.19 (m, 1H), 7.25 – 7.17 (m, 2H), 7.09 (d, *J* = 7.3 Hz, 1H), 2.26 (s, 1H), 2.12 (s, 1H), 1.94 (dt, *J* = 20.2, 9.8 Hz, 2H), 1.76 (s, 2H), 1.60 (s, 1H), 1.45 – 1.34 (m, 1H), 1.31 (d, *J* = 9.4 Hz, 3H).
LC/MS (APSI) m/z [M+H] calculated for C20H22FN4O2: 369.2; found: 369.4.

References

1. Nakao, K. *et al.* CJ-023,423, a novel, potent and selective prostaglandin EP4 receptor antagonist with antihyperalgesic properties. *J. Pharmacol. Exp. Ther.* **322**, 686–694 (2007).
2. Murase, A. *et al.* Effect of prostanoid EP4 receptor antagonist, CJ-042,794, in rat models of pain and inflammation. *Eur. J. Pharmacol.* **580**, 116–121 (2008).
