# Supplementary material for: Docking for EP4R antagonists active against inflammatory pain
Source: Nat Commun. 2023 Dec 6;14:8067. doi: 10.1038/s41467-023-43506-6 (PMC10700596; doi:10.1038/s41467-023-43506-6)
Supplement: Supplementary file 3 — Description of Additional Supplementary Files [file 41467_2023_43506_MOESM3_ESM.pdf]

Description of Additional Supplementary Files

File Name: **Supplementary Data 1**

Description: List of all compounds described in this work.
For each compound, the compound number, the virtual library code (ZINC / Design ID), the Enamine catalog code (Enamine catalog ID), 2D information (SMILES) and a brief description whether the compound was derived from virtual screening or optimization of initial hits (Set) are provided. For virtual screening compounds, the displacement of radiolabeled 3HPGE2 at 10 µM in the initial binding assay at EP4R is provided.
For hit compounds, logIC50, IC50 and Ki in the EP4R PRESTO-Tango or EP4R Arrestin-BRET assays are shown. For lead compounds, the Kb in the EP4R cAMP Assay and Ki from the EP4R radioligand displacement assay are listed.

File Name: **Supplementary Data 2**

Description: Off-target screening of compound 74 against a panel of 97 human kinases (KINOMEscan, Eurofins item 87-0002-1000). Compound 74 was screened at 10 µM.

File Name: **Supplementary Data 3**

Description: Input, parameter, and output files from molecular dynamics simulations.
