# Supplementary material for: Docking for EP4R antagonists active against inflammatory pain
Source: Nat Commun. 2023 Dec 6;14:8067. doi: 10.1038/s41467-023-43506-6 (PMC10700596; doi:10.1038/s41467-023-43506-6)
Supplement: Supplementary file 7 — Reporting Summary [file 41467_2023_43506_MOESM7_ESM.pdf]

nature portfolio

Corresponding author(s): Allan I Basbaum, Bryan L Roth, Brian K Shoichet

Last updated by author(s): October 20, 2023

Reporting Summary

Nature Portfolio wishes to improve the reproducibility of the work that we publish. This form provides structure for consistency and transparency in reporting. For further information on Nature Portfolio policies, see our Editorial Policies and the Editorial Policy Checklist.

Statistics

For all statistical analyses, confirm that the following items are present in the figure legend, table legend, main text, or Methods section.

n/a	Confirmed	
☐	☒	The exact sample size (*n*) for each experimental group/condition, given as a discrete number and unit of measurement
☐	☒	A statement on whether measurements were taken from distinct samples or whether the same sample was measured repeatedly
☐	☒	The statistical test(s) used AND whether they are one- or two-sided<br>*Only common tests should be described solely by name; describe more complex techniques in the Methods section.*
☒	☐	A description of all covariates tested
☐	☒	A description of any assumptions or corrections, such as tests of normality and adjustment for multiple comparisons
☐	☒	A full description of the statistical parameters including central tendency (e.g. means) or other basic estimates (e.g. regression coefficient) AND variation (e.g. standard deviation) or associated estimates of uncertainty (e.g. confidence intervals)
☐	☒	For null hypothesis testing, the test statistic (e.g. *F*, *t*, *r*) with confidence intervals, effect sizes, degrees of freedom and *P* value noted<br>*Give P values as exact values whenever suitable.*
☒	☐	For Bayesian analysis, information on the choice of priors and Markov chain Monte Carlo settings
☒	☐	For hierarchical and complex designs, identification of the appropriate level for tests and full reporting of outcomes
☒	☐	Estimates of effect sizes (e.g. Cohen's *d*, Pearson's *r*), indicating how they were calculated

Our web collection on statistics for biologists contains articles on many of the points above.

Software and code

Policy information about availability of computer code

Data collection	For all in vivo behavioral tests, the experimenter was always blind to treatment.
Data analysis	DOCK3.7, Qnifft 2.2, Corina v4.2.0026, Jchem v19.15.0, Omega v.2.5.14, AMSOL v.7.1, UCSF Chimera (v1.14), Pymol (v.2.1), RDKit, GraphPad Prism v9.0, ChemDraw 20.0, MODELLER27-9v15, GROMACS 2018, martinize.py, insane.py, Microsoft Excel v16.77.1

For manuscripts utilizing custom algorithms or software that are central to the research but not yet described in published literature, software must be made available to editors and reviewers. We strongly encourage code deposition in a community repository (e.g. GitHub). See the Nature Portfolio guidelines for submitting code & software for further information.

Data

Policy information about availability of data

All manuscripts must include a data availability statement. This statement should provide the following information, where applicable:

- Accession codes, unique identifiers, or web links for publicly available datasets
- A description of any restrictions on data availability
- For clinical datasets or third party data, please ensure that the statement adheres to our policy

The ZINC compound library is available to all at https://zinc20.docking.org/ and https://zinc15.docking.org/. The additionally generated anion library is freely available at https://ep4.docking.org/. Most if not all molecules from the anion library are also available in the ZINC-22 compound library (https://cartblanche22.docking.org/). The PDB entry for the EP4R crystal structure used for docking calculations is 5YWY (https://www.rcsb.org/structure/5ywy). Figures

with associated raw data include Figures 1, 2 and 3, and Supplementary Figures 4, 5, 6, 7, 9, and 10. Source data are provided with this paper. All de novo compounds are listed in Supplementary Data set 1. Off-target screening results of the lead compound against a panel of 97 human kinases is provided in Supplementary Data set 2. Chemical identities, purities (LC/MS), yields and spectroscopic analysis (H-NMR) for active compounds are provided in Supplementary Methods. Input, parameter, and output files of molecular dynamics simulations are provided with this paper. All other data is available upon request.

Research involving human participants, their data, or biological material

Policy information about studies with human participants or human data. See also policy information about sex, gender (identity/presentation), and sexual orientation and race, ethnicity and racism.

Reporting on sex and gender	No human participants, data or biological material was used in this study.
Reporting on race, ethnicity, or other socially relevant groupings	No human participants, data or biological material was used in this study.
Population characteristics	No human participants, data or biological material was used in this study.
Recruitment	No human participants, data or biological material was used in this study.
Ethics oversight	No human participants, data or biological material was used in this study.

Note that full information on the approval of the study protocol must also be provided in the manuscript.

Field-specific reporting

Please select the one below that is the best fit for your research. If you are not sure, read the appropriate sections before making your selection.

☒ Life sciences ☐ Behavioural & social sciences ☐ Ecological, evolutionary & environmental sciences

For a reference copy of the document with all sections, see nature.com/documents/nr-reporting-summary-flat.pdf

Life sciences study design

All studies must disclose on these points even when the disclosure is negative.

Sample size	For in vivo behavioral studies, we did not perform sample-size calculations. We modeled our sample sizes for behavioral studies on previous studies that used a similar approach to our own, which were capable of detecting significant changes. Similar studies were performed e.g. in Fink et al., Science, 2022 (doi: 10.1126/science.abn7065). For in vitro characterization of molecules we did not perform sample-size calculations. Single concentration screening of initial docking hits using 3H-PGE2 displacement assay was performed as one biological replicate with n=3 (compounds from first docking screen) or n=4 (compounds from second docking screen) technical replicates (see Extended dataset 1). The GPCRome was performed as one biological experiment with n=4 technical replicates. Full dose-response curves shown throughout this manuscript were typically derived from three biological replicates. All sample sizes are described in figure legends.
Data exclusions	No data were excluded.
Replication	Full dose-response curves obtained from BRET, TANGO and radioligand competitive binding assays, were performed in three biologically independent experiments. All sample sizes are provided in figure legends. Single concentration screening of initial docking hits using 3H-PGE2 displacement assay was performed as one biological replicate with n=3 (compounds from first docking screen) or n=4 (compounds from second docking screen) technical replicates (see Extended dataset 1). The GPCRome was performed as one biological experiment with n=4 technical replicates. For in vivo studies, replications were performed with the number of individual mice that were used in each experiment. All attempts at replication were successful.
Randomization	Male C56BL/6 mice were randomly assigned to the treatment group and control group. Animals were initially placed into one cage and allowed to free run for a few minutes. Next, each animal was randomly picked up, injected with the drug or vehicle control and placed into a separate cylinder before the behavior test. For all in vitro characterization of docking hits randomization is irrelevant as no group allocations were performed.
Blinding	The experimenters who conducted the in vivo experiments were blinded to treatment conditions of the mice. For functional in vitro studies, blinding is not necessary since these experiments do not require subjective assessment of the data by the experimenter.

Reporting for specific materials, systems and methods

We require information from authors about some types of materials, experimental systems and methods used in many studies. Here, indicate whether each material, system or method listed is relevant to your study. If you are not sure if a list item applies to your research, read the appropriate section before selecting a response.

Materials & experimental systems

n/a	Involved in the study	
☒	☐	Antibodies
☐	☒	Eukaryotic cell lines
☒	☐	Palaeontology and archaeology
☐	☒	Animals and other organisms
☒	☐	Clinical data
☒	☐	Dual use research of concern
☒	☐	Plants

Methods

n/a	Involved in the study	
☒	☐	ChIP-seq
☒	☐	Flow cytometry
☒	☐	MRI-based neuroimaging

Eukaryotic cell lines

Policy information about cell lines and Sex and Gender in Research

Cell line source(s)	HTLA cells stably expressing a tTA-dependent luciferase reporter and a beta-arrestin2-TEV fusion gene were a gift from the laboratory of R. Axel (Columbia University). HEK293T cells were obtained from the American Type Culture Collection (ATCC, CRL-11268).
Authentication	HTLA cells were authenticated by morphology and growth characteristics, and the successful TANGO assay which demonstrates that both tTA-dependent luciferase reporter and a β-arrestin2-TEV fusion gene are present in the cells. HEK293T cells were authenticated by the supplier (ATCC) using morphology and growth characteristics and STR profiling.
Mycoplasma contamination	HEK293T cells lines tested negative for mycoplasma contamination (Hoechst DNA strain and Direct Culture methods employed). HTLA cells were not in particular tested for mycoplasma contamination.
Commonly misidentified lines (See ICLAC register)	No commonly misidentified cell lines were used in this study.

Animals and other research organisms

Policy information about studies involving animals; ARRIVE guidelines recommended for reporting animal research, and Sex and Gender in Research

Laboratory animals	For Pharmacokinetic studies: Species: Mus musculus; Genotype: CD-1; sex: males; age: 9 weeks. Mice were housed in cages on a standard 12:12 hour light/dark cycle at 22 degrees celsius and relative humidity of 40 - 70%. For behavioral studies: Species Mus musculus; Genotype: C56BL/6; sex: males; age: 8-10 weeks. Mice were housed in cages on a standard 12:12 hour light/dark cycle with food ad water ad libitum, at 22 degrees celsius and relative humidity of 40%.
Wild animals	No wild animals were used in this study.
Reporting on sex	All in vivo experiments were conducted in male mice. Sex was not considered in the study design and is not considered to significantly change study outcomes.
Field-collected samples	No field collected samples were used in this study.
Ethics oversight	Animal behavioral experiments were approved by the UCSF Institutional Animal Care and Use Committee and were conducted in accordance with the NIH Guide for the Care and Use of Laboratory animals (protocol #AN195657). Animal pharmacokinetic studies were in accordance with the Enamine PK study protocols and Institutional Animal Care and Use Guidelines.

Note that full information on the approval of the study protocol must also be provided in the manuscript.
